# Exploring dynamical whole-brain models in high-dimensional parameter spaces

**DOI:** 10.1371/journal.pone.0322983

**Published:** 2025-05-12

**Authors:** Kevin J. Wischnewski, Florian Jarre, Simon B. Eickhoff, Oleksandr V. Popovych

**Affiliations:** 1 Institute of Neuroscience and Medicine – Brain and Behaviour (INM-7), Forschungszentrum Jülich, Germany; 2 Institute of Systems Neuroscience, Medical Faculty and University Hospital Düsseldorf, Heinrich Heine University Düsseldorf, Germany; 3 Institute of Mathematics, Faculty of Mathematics and Natural Sciences, Heinrich Heine University Düsseldorf, Germany; Western University, CANADA

## Abstract

Personalized modeling of the resting-state brain activity implies the usage of dynamical whole-brain models with high-dimensional model parameter spaces. However, the practical benefits and mathematical challenges originating from such approaches have not been thoroughly documented, leaving the question of the value and utility of high-dimensional approaches unanswered. Studying a whole-brain model of coupled phase oscillators, we proceeded from low-dimensional scenarios featuring 2–3 global model parameters only to high-dimensional cases, where we additionally equipped every brain region with a specific local model parameter. To enable the parameter optimizations for the high-dimensional model fitting to empirical data, we applied two dedicated mathematical optimization algorithms (Bayesian Optimization, Covariance Matrix Adaptation Evolution Strategy). We thereby optimized up to 103 parameters simultaneously with the aim to maximize the correlation between simulated and empirical functional connectivity separately for 272 subjects. The obtained model parameters demonstrated increased variability within subjects and reduced reliability across repeated optimization runs in high-dimensional spaces. Nevertheless, the quality of the model validation (goodness-of-fit, GoF) improved considerably and remained very stable and reliable together with the simulated functional connectivity. Applying the modeling results to phenotypical data, we found significantly higher prediction accuracies for sex classification when the GoF or coupling parameter values optimized in the high-dimensional spaces were considered as features. Our results elucidate the model fitting in high-dimensional parameter spaces and can contribute to an improved dynamical brain modeling as well as its application to the frameworks of inter-individual variability and brain-behavior relationships.

## Introduction

Over the past years, neuroimaging studies have intensively been striving to seize the full potential of mathematical models designed for the simulation of human brain dynamics. Especially the investigations of the resting-state brain activity [1] involved a plenitude of dynamical whole-brain models. These models incorporate anatomical information about the brain into the simulation of its dynamical properties either on the group or subject level [2–5]. While anatomical information in this context refers to structural connectivity (SC), i.e., physical connections in terms of axonal bundles between brain regions, dynamical properties are thought of as functional connectivity (FC), i.e., the amount of temporal coactivations between regions. SC and FC show a complex and relatively weak relationship that also depends on the considered brain parcellation [6–8]. Data-driven dynamical models equip the structural data with dynamical features [9] and can therewith shed light onto the mechanisms underlying the emergence of resting-state brain dynamics [2,10–13]. Additionally, model simulations may improve the overall interpretability by performing a certain denoising, meaning that only the relevant dynamics and phenomena are modeled, but not the unwanted effects of random noise present in the empirical data (or at least the impact can be controlled and investigated) [2,3,14–16].

As model simulations can be designed to reproduce resting-state dynamics on a personalized level, researchers strive to link the empirical observations to the ingredients and dynamical properties of the computational model [17]. That can possibly be done by approximating optimal model parameters, leading to a better mechanistic explanation of the observed brain activity [18,19]. In line with that, recent studies have reported an improved differentiation of Alzheimer’s disease (AD) [20] and facilitated identification of new attention deficit hyperactivity disorder (ADHD) subtypes [21] based on model parameters of a reduced Wong-Wang model and a coupled oscillator model, respectively, and higher classification accuracy of Parkinson’s disease (PD) patients versus healthy controls [22] on the basis of connectomes simulated via a reduced Jansen-Rit model to give a few examples. Personalized virtual brain models could also assist at the estimation of the epileptogenic zone and simulation of the virtual neurosurgery [23].

However, the precondition for all mentioned advantages and applications is a proper model validation against empirical data. Model validation is also referred to as model fitting or model inversion [24–27]. The approximation of optimal model parameters remains a challenge because a complete parameter space scan on a dense grid (grid search) poses exponentially growing computational requirements for every single subject. Simulations introducing region-specific model parameters, i.e., parameter heterogeneity, may however involve more than 100 free parameters [27,28]. In such cases, a grid search is unfeasible, which constitutes a major limiting factor for the aspirations of personalized brain modeling mentioned above.

Several works were already focusing on the investigation of regional heterogeneity [28–31]. However, only a few model parameters were actually optimized. By incorporating anatomical information in form of the myelin content [29], gene expression profiles [30] or the T1-weighted/T2-weighted ratio [31] into the model, a function can be derived from empirical brain imaging data that returns the parameter values of every brain region. This function in turn depends on only a few free global parameters (6 [29] or 3 [30], for example), which greatly simplifies a genuine regional parameter heterogeneity and does not require its optimization in high-dimensional parameter spaces. Similarly, Kong and colleagues [28] suggested to constrain the regional heterogeneity of a parametric mean-field model by both anatomical and functional gradients and therefore reduced their original model featuring 205 free parameters to a simpler one with only 10.

The precise replication of personalized resting-state dynamics may nevertheless require the transition to high-dimensional model parameter spaces [18,23,27]. In this scenario, optimal parameters can comprise degenerate manifolds that ideally would be sampled systematically by Monte Carlo approaches [23,29,32–34]. With respect to computational resource consumption and tractability, however, the more practical solution is often given by the use of mathematical optimization algorithms [28,35–40]. These methods suggest new sampling points iteratively and aim at convergence to an optimal parameter configuration [41,42].

In this study, we investigate the behavior of a whole-brain model when the model fitting to subject-specific empirical data is performed in a high-dimensional parameter space. We illustrate this on a simple model of coupled phase oscillators when the similarity between simulated and empirical FC was maximized by simultaneous optimization of about 100 model parameters via two dedicated optimization algorithms: Bayesian Optimization (BO) and the Covariance Matrix Adaptation Evolution Strategy (CMAES). The latter were selected as efficient approaches for model validation in low-dimensional parameter spaces in our previous study [42]. Our ambition in the current study was to fully exploit the algorithms’ superiority over a grid search [42] and explore the benefits and challenges that arise with real high-dimensional parameter spaces. We wanted to assess the practical value for more precise replications of the resting-state brain dynamics and possible relations to phenotypical data as additional values for the application and validation of the modeling results. This may contribute to the existing literature on brain modeling and show that the mathematical methods allow us to validate whole-brain models that feature a genuine, unconstrained parameter heterogeneity.

Below, we demonstrate that the high-dimensional model validation can indeed strongly improve the quality of the model fitting with a respective (moderate) increase of the required computational resources. We also inspect and document the properties of the optimized model parameters, the corresponding matrices of simulated FC (sFC) as well as their reliability and potential utility for further applications. Despite persistent fluctuations within the approximated optimal model parameters, the sFC remains relatively stable. Finally, we take phenotypical data of individual subjects into account and analyze the impact of the parameter space’s dimension on the extent of differences between males and females observed in simulated data. We find enhanced sex differences in the modeling results for high-dimensional parameter spaces, leading to significantly higher classification accuracies than for the low-dimensional model fitting, which can serve as further indications of the applicability and validity of the employed modeling approaches and reported results.

## Methods

In the present study, we considered neuroimaging data of S = 272 healthy, unrelated subjects (age 28.5 ± 3.5 years, 128 males) from the Human Connectome Project (HCP) S1200 public dataset release [43]. The local ethics committee of the HCP WU-Minn gave its approval for the project, and written, informed consent was obtained from all subjects. All experiments were performed in accordance with the relevant guidelines and complied with the principles expressed in the Declaration of Helsinki.

For our investigations, the brain was parcellated (according to a given brain atlas) into separate brain regions used for the calculation of SC and resting-state FC. A simple dynamical whole-brain model of coupled phase oscillators was then applied to simulate the resting-state brain dynamics of every subject based on the individual structural data. We inferred sFC from the model output and compared it to the empirical FC (eFC). By the use of the Pearson correlation coefficient, we assessed their similarity, i.e., the quality of the model fit, and searched for model parameter values that maximize it. We will also refer to this procedure as model validation. Further, we investigated the impact of the number of free model parameters used for simultaneous optimization and therefore considered various parameter spaces for model validation.

Our goal was to extract information about the results of the model validation in the high-dimensional parameter spaces. We wanted to evaluate the properties of the optimized model parameters, sFC and the extent of similarity between empirical and simulated data. In particular, we were interested in (i) the presence of multiple optima in the high-dimensional parameter spaces and (ii) the identifiability of the simulation results, which could be affected by the convergence to different optima in repeated algorithm executions. We therefore implemented two mathematical optimization methods, BO and CMAES, to search for optimal parameter values (model validation) which put the model output in the closest possible correspondence with empirical data. After performing several repetitive model validations for different initial conditions for the same subject, we analyzed the modeling outcomes with respect to their variability, reliability and practical utility. Finally, we inspected the applicability of the modeling results to phenotypical data. We were especially interested in how much high-dimensional model validation can reveal sex differences in the modeling results, compared to the cases of low-dimensional model fitting as well as purely empirical brain imaging data.

### Empirical and simulated data

#### MRI data preparation.

The considered HCP dataset included complete T1-weighted and diffusion-weighted magnetic resonance imaging (dwMRI) as well as resting-state functional MRI (fMRI) data for every subject. The extraction of SC was performed with a publicly available pipeline (https://github.com/inm7/vbc_dwmri). We refer to our related works [8,44] for further details about the utilized software tools. The reconstructed, parcellation-based SC comprised two matrices: one featuring the number of streamlines between individual regions, i.e., the streamline count, henceforth referred to as SC, and another one providing the average length of streamlines between regions, termed path lengths (PL). We considered the functional Schaefer atlas [45] with N = 100 cortical regions (Sch100) as well as the anatomical Harvard-Oxford atlas [46] featuring N = 96 regions and a probability threshold of 0% (HO0Thr).

The calculation of eFC in turn was based on the resting-state fMRI data provided by the HCP repository [43]. Time series of the blood-oxygen-level-dependent (BOLD) fluctuations were extracted as mean signals averaged over all voxels of individual brain regions parcellated according to the considered brain atlases. A subsequent calculation of the Pearson correlation for each pair of linearly detrended and z-scored BOLD time series from the respective cortical areas then yielded the desired eFC matrix. In this setting, four fMRI sessions comprising 1200 volumes each (sampled with a repetition time of TR = 0.72 s) were available for every subject. Scans were performed twice using different phase-encoding directions on two days. A concatenated BOLD signal was generated by combining the z-scored BOLD time series from the mentioned sessions. We focused on the eFC matrix derived from this concatenation (and eventual cross-correlation of the regional signals) for the optimization of model parameters in terms of the best fit between simulated and empirical data, i.e., between sFC and eFC.

#### Model simulations.

To obtain sFC, we implemented the Kuramoto model of coupled phase oscillators [47–49]. In the model, each of N oscillators represented one brain region defined by the underlying parcellation (N ∈ {96, 100} in this study). Their temporal dynamics served as a basis to compute simulated BOLD signals that were then cross-correlated in order to derive sFC. More precisely, the phase dynamics of the mean BOLD signal of a brain region i ∈ {1, …, N} can be described by the following differential equation:


$${\overset{˙}{\theta }}_{i}(t)=2\pi f_{i}+\sum_{j=1}^{N}k_{ij}\text{sin}\left(\theta_{j}\left(t-\tau_{ij}\right)-\theta_{i}(t)\right)+\sigma \eta_{i}(t).$$ (1)

For a given time t, $\theta_{i}(t)$ denotes the phase of the $i^{\text{th}}$ oscillator that oscillates with a natural frequency ${2\pi f}_{i}$ if left uncoupled ($k_{ij}$ = 0) from the other oscillators. This assumption considers that the ensemble of neurons contained within a cortical area behaves coherently, leading to a well-defined average phase, whose dynamics is described by the natural frequency [50,51]. For the low-dimensional cases, the frequency parameters $f_{1}$, …, $f_{N}$ were estimated from the maximal spectral peaks (restricted to the frequency range from 0.01 Hz to 0.1 Hz) of the empirical BOLD time series. In the model simulations and validations with high-dimensional parameter spaces in turn, we treated them as free parameters restricted to the same range.

Apart from intrinsic oscillations, the phase dynamics is influenced by delayed interactions with the other oscillators. The individual coupling strengths $k_{ij}$ and delay values $\tau_{ij}$ were derived from the empirical matrices $\begin{array}{c} \text{SC}={\left({\text{SC}}_{ij}\right)}_{1\leq i,j\leq N} \end{array}$ and $\begin{array}{c} \text{PL}={\left({\text{PL}}_{ij}\right)}_{1\leq i,j\leq N} \end{array}$, respectively:


$$k_{ij}=\frac{{\text{SC}}_{ij}}{\left\langle \text{SC}\right\rangle }\frac{C}{N},{\;\tau }_{ij}=\frac{{\text{PL}}_{ij}}{\left\langle \text{PL}\right\rangle }\tau .$$ (2)

In this context, $\begin{array}{c} \left\langle \;\;\;.\right\rangle \end{array}$ indicates the mean over all matrix elements, excluding the diagonals. C and τ are global weighting factors that influence the strength and delay of the interactions between individual oscillators, respectively. These quantities were treated as free parameters in all considered scenarios. Another variable for optimization can be the intensity σ of the independent noise $\eta_{i}(t)$ which is sampled from a uniform distribution over [-1, 1] and perturbs the individual oscillators. While C and σ are dimensionless quantities, τ is measured in seconds.

We solved Eq. (1) numerically using Heun’s method for stochastic differential equations [52] with a discrete time step of Δt = 0.06 s. Here, we simulated phase dynamics for 3500 s after skipping 500 s as a transient. The calculated phases were down-sampled to 0.72 s in order to match the repetition time of the fMRI data provided by the HCP. With the model at hand, we generated simulated BOLD time series by computing $\text{sin}\left(\theta_{i}(t)\right)$ for all i ∈ {1, …, N} corresponding to the regions in the respective brain parcellation. As mentioned above, sFC was then derived from the Pearson correlation coefficients across all pairs of simulated time series. We assessed the similarity between simulated and empirical data by correlating the matrices of sFC and eFC, where the former depended on the model parameters C, τ, σ and ${\left(f_{i}\right)}_{1\leq i\leq N}$.

#### Parameter optimization.

As mentioned above, the model was validated by optimization of the model parameters for the best correspondence between sFC and eFC. The case, where the two model parameters of global coupling and delay were optimized, corresponds to the model validation in a two-dimensional (2D) parameter space, see also [49,53]. Besides, a three-dimensional (3D) parameter space was considered, where also the noise intensity was treated as a free parameter, see also [3]. These two scenarios can be described as the low-dimensional cases. In our preceding work [42], we performed a systematic comparison of optimization methods applied to individualized whole-brain models in such low-dimensional parameter spaces (for fewer subjects than here and only one brain atlas). After investigating the stability and computational expenses of several approaches, we identified the best performing algorithms (BO, CMAES) for further usage. Here, we additionally worked with two high-dimensional parameter spaces for two brain atlases, in which we adapted the idea of region-specific model parameters that had already been motivated in [27], for example. We therefore applied our previously tested methods, circumvented the unsurmountable resource requirements of a potential grid search and optimized the frequency parameters for all brain regions. This extends previous attempts to introduce regional heterogeneity, in which the natural frequencies in the Kuramoto model were based on anatomical node strengths, i.e., row sums in the SC matrix [54–56]. Taking into account the three previously mentioned parameters of global coupling, delay and noise intensity, this ultimately led to 99-dimensional (99D) and 103-dimensional (103D) parameter spaces for the Harvard-Oxford atlas and the Schaefer atlas, respectively. We note here that the consideration of local model parameters for all brain regions appeared as the most logical step for introducing regional heterogeneity in the considered phase oscillator model.

Applying two mathematical optimization methods instead of one appeared reasonable since we could not always use a grid search as a baseline for result verification. Our confidence in both methods was based on previous successful applications and analyses [28,42,57–59]. In the current study, however, we did not intend to compare the methods as such very extensively. We treated them more as a mutual reassurance to minimize the risk of biased result interpretations due to algorithm peculiarities. Instead, we inspected the differences between models validated in low- and high-dimensional parameter spaces. We therefore investigated the approximated optimal parameters and obtained fitting quality together with the sFC and computational requirements in all tested dimensions.

We repeat at this point that we worked with several model parameter spaces. In the simplest case (2D), we fixed the noise intensity at σ = 0.3 and extracted the frequency parameters $f_{1}$, …, $f_{N}$ from the empirical BOLD signals. The free parameters were then the global coupling strength C ∈ [0, 1] and the global delay τ ∈ [0, 100]. In the 3D scenario, we increased the dimension of the parameter space by adding σ ∈ [0, 2] as a free variable. The motivation of the considered parameter ranges and the efficacy of the considered optimization algorithms on such problems are detailed in our preceding work [42]. For the high-dimensional simulations, which constitute the essence of this study, we further included all remaining free parameters of the model, namely the natural frequencies $f_{1}$, …, $f_{N}$ ∈ [0.01, 0.1] as parameters for simultaneous optimization. This resulted in the 99D and 103D parameter spaces for HO0Thr and Sch100, respectively. The range for C was increased to [0, 2] here in order to enable a more global search in the high-dimensional cases.

All model fitting simulations for this study were performed on the CPU partition of the high-performance supercomputing cluster JURECA-DC at Forschungszentrum Jülich. Every computation node featured two AMD EPYC Rome 7742 processors with 64 cores each, and each core supported two hardware threads, so that one computation node ultimately offered 2 × 2 × 64 = 256 threads for simultaneous computations, see [60] for further details about the configuration of JURECA-DC.

#### Optimization algorithms.

The BO method is a sequential design strategy suited for optimization problems with an unknown goal function F that is expensive or time-consuming in its evaluation. After computing the goal function values for a random sample of Λ ∈  N  candidate solutions in the parameter space, the obtained information is used to construct a probabilistic surrogate model for F [61]. This approximation of F can be updated based on the knowledge obtained from further function evaluations. Ideally, the surrogate model will eventually deliver a close approximation of the goal function, thereby enabling its efficient optimization. Detailed descriptions of all relevant steps can be found in algorithm-related papers [42,62,63]. We used the C++ software package *BayesOpt* [64] for our simulations. After analyzing the gradual development of the correlation between sFC and eFC for a saturation (depending on the number of performed iterations), we applied a stopping criterion in the form of a maximal number of iterations. We worked with 80 and 230 iteration steps in the low- and high-dimensional cases, respectively, see also Supplementary Materials. For every subject and parameter space, we performed $R_{\text{max}}$ = 30 executions of BO with random initial data. Some results of the convergence tests are shown in **Supplementary Fig 1** in S1 Appendix .

The CMAES constitutes a global, population-based search procedure. In every iteration, a sample of Λ candidate solutions is drawn from a multivariate normal distribution. We refer to it as the *search distribution*. CMAES works with a weighted mean of $\lfloor \frac{\Lambda }{2}\rfloor$ solutions which yield the lowest values of the goal function (in a minimization problem). Afterwards, a new generation of candidates is obtained by taking Λ new samples from the search distribution centered around the weighted mean of these most promising points. That is how the search distribution is adapted iteratively towards a concentration around the optimal solutions, see [42,65,66] for more details. In accordance with the setting for BO, we also executed CMAES $R_{\text{max}}$ = 30 times for every subject and parameter space, see also Supplementary Materials. The initial search distribution mean was selected uniformly randomly within the considered parameter ranges. The correlation of sFC and eFC showed a saturation after 80 and 150 iteration steps in the low- and high-dimensional parameter spaces, respectively. Some examples of the performed convergence tests are shown in **Supplementary Fig 1** in S1 Appendix.

### Analysis of modeling results

As indicated above, we assessed the quality of the model fit by computing the Pearson correlation coefficient between the matrices of sFC and eFC, where the former depended on the selected model parameters. The highest correlation between such two matrices (found by the optimization of the model parameters) was termed the model’s *goodness-of-fit* (GoF). We collected and analyzed the approximated optimal model parameters as well as the respective GoF values and sFC matrices for each of 30 independent runs of the optimization methods for all subjects and parcellations. In particular, we analyzed the test-retest reliability of the optimized model parameters, GoF values and sFC edges, for which we defined reliability as the intraclass correlation (between-subject variance relative to total variance [67]). Hereby, we deliberately deviated from the classical procedure of considering the variability within subjects across repeated empirical measurements (test-retest) and worked with the variability across repeated optimization algorithm initializations instead. Our intention was to see how variable the modeling output is across independent executions of the validation procedure for the same subject as well as between subjects. Particular attention was devoted to the high-dimensional cases.

#### Intra-subject variability of approximated optimal frequency parameters.

We stress that we had added the frequencies $f_{1}$, …, $f_{N}$ to the list of parameters for optimization in the high-dimensional cases (99D, 103D). Due to such an increase in the dimension of the parameter space and the related complexity of the corresponding model validation procedure, we had to elaborate more on the properties of the optimized model parameter values. Besides the optimized triple (C, τ, σ), we obtained N optimized frequency values ${\left(f_{i}\right)}_{1\leq i\leq N}$ after every algorithm execution with random initial conditions for a given subject. This means that in addition to the three global parameters of delay, coupling and noise intensity, the simulations yielded specific local parameters related to every brain region. An analysis of the distributions and structure of the latter parameters was performed to inspect the variability within subjects and check for potential subject-specific characteristics in the optimized values. In search of such common patterns across any two executions

$q_{1},q_{2}$ ∈ {1, …, $R_{\text{max}}$}, $q_{1}\neq q_{2}$, we correlated all pairs of vectorized frequency sets ${\left(f_{i}\right)}_{1\leq i\leq N}^{q_{1}}$ and ${\left(f_{i}\right)}_{1\leq i\leq N}^{q_{2}}$. For every subject s ∈ {1, …, S}, we then saved the obtained correlation values to a matrix $A_{s}$ ∈ ${\mathbb{R}}^{R_{\text{max}}\times R_{\text{max}}}$ and applied the k-means clustering method [68] to the rows of $A_{s}$. Additionally, we computed the sum of all squared distances between the cluster elements and their cluster centroids. The final number of clusters k was then selected by the “elbow method” as the one for which the sum of distances showed the strongest decrease as compared to the previous case $\begin{array}{c} k\;- \end{array}$ 1, and where the larger number of clusters $\begin{array}{c} k\;+ \end{array}$ 1 did not reduce it notably. As a quantification of the cluster size, we considered the number of elements in the largest cluster. The number and size of clusters were however set to zero if the absolute mean of the off-diagonal elements in the correlation matrix $A_{s}$ was below 0.25.

As an extension to the clustering procedure, we also investigated whether a principal component analysis (PCA) of the obtained model parameters can provide additional insights about the structure of the solutions.

#### Reliability of modeling outcomes.

We analyzed the test-retest reliability of the simulation results with the intraclass correlation coefficient (ICC) [67,69–71]. In this approach, we considered the optimized model parameters and the obtained GoF values as well as the elements/edges of the sFC matrices. The ICC quantifies the between-subject variance of the mentioned values relative to the total variance, i.e., between and within subjects. As stated above, we treated repeated executions of the optimization algorithms as proxies for different scanning sessions. Higher ICC scores imply more reliable results.

There exist many different definitions of the ICC in the literature, all of them being suitable for particular problems [67]. We worked with the ICC that is also known as ICC(1) or ICC(1,1) and can be described as the original ICC without bias or systematic error [67]. It is defined as follows:


$$\text{ICC}=\frac{o_{\text{subject}}^{2}}{o_{\text{subject}}^{2}+o_{\text{residual}}^{2}}$$ (3)

where $o_{\text{subject}}^{2}$ denotes the variance of the considered quantity that is related to the variance among subjects and $o_{\text{residual}}^{2}$ represents the remaining residual variance that may be induced by different measurements of a given subject, for instance. It has been proposed to use this definition in cases where the sources of fluctuations within subjects are unspecified and no structured variability can be assumed [70,71]. This was adequate for our setting since we only changed the random initial data of every algorithm execution for a given subject. To compute the ICC, we used the approximative sample ICC recommended in [67]. It allowed us to inspect the ratio of the fluctuations of the simulation outcomes between subjects relative to the fluctuations between algorithm executions across and within subjects. We adapted the quality gradation suggested in [72,73], see also Supplementary Materials.

As an extension of the analyses pertaining to the fluctuations within and between subjects, we also computed the *subject specificity* [74–76] derived from the differences between intra- and inter-subject (i) FC correlations and (ii) FC matrix norm distances. The details are provided in the Supplementary Materials. GoF-based specificity measures were computed as well, see also Supplementary Materials.

### Relations to phenotypical data

As mentioned above, the cohort in this study consisted of S = 272 healthy subjects, with 128 males and 144 females. Our intention was also to extend the application of the modeling results beyond neuroimaging data, and we investigated phenotypical differences in the simulated data. After controlling our results for GoF outliers, we repetitively selected a random execution number $q_{s}$ ∈ {1, …, $R_{\text{max}}(s)$}, $R_{\text{max}}(s)$ ≤ 30, separately for every subject s ∈ {1, …, S} and subsequently defined a vector u ∈ ${\mathbb{R}}^{S}$ containing the corresponding optimal parameter or GoF values detected in these algorithm executions. We then split the data of males and females and computed the effect size (ES, see next section) of the differences in the parameter or GoF values. This procedure was repeated 1000 times for all parameter spaces and optimization methods, so that we ultimately obtained 1000 estimates of the actual ES. We wanted to see whether the simulation outcomes could reveal pronounced sex differences, which appeared to us as the most suited initial practical application and validation of our considered modeling approach.

When investigating the phenotypical differences in the simulated data, we took into account that the intracranial brain volume (brain size) usually tends to be larger for males than for females [77]. We therefore regressed out the brain size from the simulation results like GoF and also assessed the sex differences in the residuals. Such an analysis can provide further insights if the sex differences observed in the simulation results were caused by the differences in brain size.

As a purely empirical alternative to the simulated data, we also briefly inspected the ES of the differences in the correlations between SC and eFC and compared them to the values that we observed for the modeling results.

To bolster the practical utility of our results in case of fluctuating solutions, we further analyzed suited alternatives to select *one* final outcome per subject out of the 30 available solutions of the algorithm executions. We therefore compared the results for the selection of the algorithm runs yielding

- the highest GoF (option 1),
- the median GoF (option 2),
- the minimal ${\left\|\text{sFC}-\text{eFC}\right\|}_{\text{F}}$ (option 3),

where ${\left\|.\right\|}_{\text{F}}$ denotes the Frobenius norm, see Supplementary Materials. For the optimization problem considered in this work, these three options represent mathematically intuitive selection strategies. Note that our models were fitted to maximize the correlation between sFC and eFC, but not to minimize the matrix norm of their difference.

For the mentioned alternatives, we analyzed the extent of sex differences in the FC edges (above the diagonal) and also computed balanced accuracies [22] for a machine learning sex classifier being trained on either the GoF values or the optimized model parameters.

For the sex classification, we resorted to a publicly available classification script developed at our lab (https://github.com/kyesam-jung/model-based-prediction/). It applied a nested 5-fold cross-validation (CV) scheme, where every outer CV loop included five embedded inner loops as a nested CV (inner 5-fold CV) for training the prediction model (a logistic regressor, see below). The training procedure started with a random splitting of the entire subject cohort into five subgroups of equal size, while maintaining the ratio of females and males in each subgroup. Subsequently, in every outer loop, one subgroup was selected after another as a test set, and the other four subgroups were united into a training set. In the inner loop with the training set, a confound removal (CR) was performed to eliminate the effect of brain size on the sex classification from the features, *i.e.*, GoF values or model parameters. Finally, the approach used a logistic regression with an L2 penalty for the training in the nested CV. The regularizing parameter was optimized by the *Limited memory Broyden-Fletcher-Goldfarb-Shanno algorithm* (L-BFGS) [78]. After the training in the nested CV, the best model was selected and applied to the test set to classify the unseen subjects as either males or females. Here, the respective CR and z-scoring with parameters obtained for the training set were applied beforehand. Such a CV-CR scheme prevents a data leakage as no information from the test set was used during the training [79]. This prediction process was repeated 100 times for different random subject splits into five subgroups. Finally, a balanced accuracy [22] was calculated using predicted probability values and target variables (male or female).

#### Statistical tests and effect sizes.

In this work, we utilized two non-parametric tests to assess statistical significance. When dealing with two related samples (e.g., the GoF for the same subjects, atlas and algorithm, but for a different dimension of the parameter space), we applied the Wilcoxon signed-rank test. In case of two independent samples (e.g., the GoF for males and females in the same parameter space), we used the Wilcoxon rank-sum test. For the latter method, we further assessed the ES of statistical differences with Rosenthal’s formula [80]:


$$\text{ES}=\frac{Z}{\sqrt{S}}$$ (4)

where Z denotes the z-score of the test statistic and S represents the total sample size (number of males + number of females, for example). We used this formula in accordance with related works [22,81] and adapted the quality gradation from *very low* to *very strong* according to [82], see also Supplementary Materials.

## Results

In this section, we present our findings with increasing complexity of involved analysis and result interpretation. We put particular emphasis on the results of the model validation in the high-dimensional parameter spaces, from which we derive the majority of novel insights. Our goal was not only to examine the mere increases in fitting quality (GoF) that constitute an important result of the model validation in a high-dimensional parameter space. In fact, we also wanted to investigate the extent of intra- and inter-subject variability of the modeling outcomes in the high-dimensional cases. We aimed to show and suggest the utility of such simulations for future applications related to phenotypical data and, also, to the link between brain and behavior. To design our flow of thoughts most effectively, we start with the “directly” observable effects which include the reporting on the detected GoF values, the invested computational resources and the approximated optimal model parameters. Then, we inspect the underlying structure of the model parameters and the corresponding matrices of sFC as well as their variability and reliability. Finally, we demonstrate how the dimension of the model parameter space may influence the extent of differences between the groups of males and females in simulated data.

### [I] Model validation outcomes

#### GoF, computation time and optimized model parameters.

**Fig 1:**
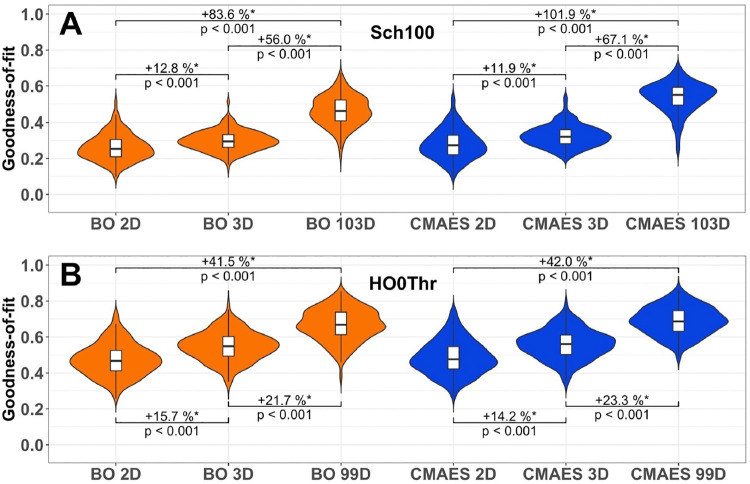
Distributions of the goodness-of-fit (GoF) values for all tested approaches and parameter spaces. The considered optimization methods and the respective dimensions of the parameter spaces are indicated on the horizontal axes (BO 2D, CMAES 2D, BO 3D and CMAES 3D in the low-dimensional cases and BO 103D, CMAES 103D, BO 99D and CMAES 99D in the high-dimensional ones) along with the detected GoF values on the vertical axes. Violins show the distributions of the median GoF values obtained for all subjects across 30 algorithm executions with random initial data (option 2, see Methods). The medians (across subjects) of the relative increase between the results obtained in different parameter spaces for a given algorithm are indicated in the plots together with p-values of the Wilcoxon signed-rank test and the considered atlas: **(A)** the Schaefer atlas (Sch100) and **(B)** the Harvard-Oxford atlas (HO0Thr). Statistically significant differences are marked with an asterisk. The significance level of 5%, p < 0.05, has been Bonferroni-corrected for multiple comparisons.

The quality of the model validation (GoF values) is influenced by the number of free model parameters (dimension of the model parameter space) being simultaneously optimized for the best correspondence between simulated and empirical data. We compared the detected GoF values across all subjects and observed that for both tested atlases, more free parameters led to a higher fitting quality [**Fig 1, Supplementary Table 2** in S1 Appendix]. Indeed, increasing the dimension from 2D to 3D resulted in a moderate median improvement of 11% – 15% [**Fig 1**] and a low ES of 0.3–0.4 for the GoF differences [**Supplementary Table 2** in S1 Appendix]. However, the transition to the high-dimensional cases caused a remarkable jump, where the median GoF could double [**Fig 1A**, CMAES for the Schaefer atlas (Sch100)]. It demonstrated a strong effect of 0.7–0.8 for the GoF differences between the 2D and high-dimensional cases for both considered atlases [**Supplementary Table 2** in S1 Appendix].

Similarly, a respective increase of required computational resources can be observed when comparing the median consumption of low- and high-dimensional optimizations [**Supplementary Fig 3** in S1 Appendix]. We however note here that more iteration steps were performed in the high-dimensional cases (see Methods and Supplementary Materials), meaning that also additional time-consuming evaluations of the goal function were made.

**Fig 2:**
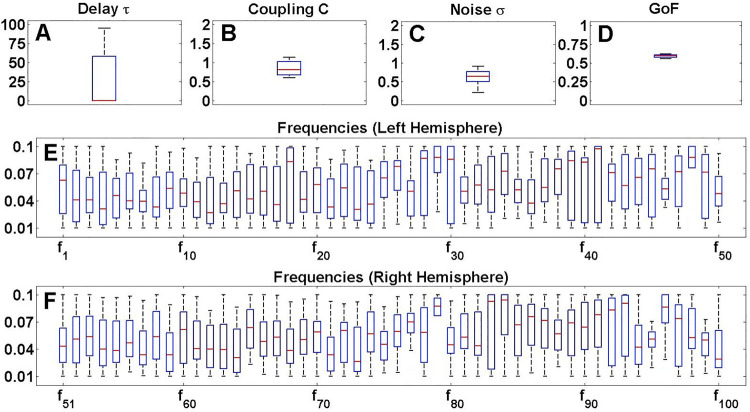
Example of the results of the model fitting in the 103-dimensional parameter space. Boxplots illustrate the distributions of the values for the optimal **(A)** delay τ, **(B)** coupling C, **(C)** noise intensity σ, **(D)** goodness-of-fit (GoF) and **(E,F)** frequency parameters ${\left(f_{i}\right)}_{1\leq i\leq N}$ found in 30 executions (with random initial data) of the CMAES algorithm for one subject. The considered modeling quantities are indicated in the titles of each plot, while their considered ranges are given on the vertical axes. Plot **(E)** shows the first half of the frequency parameters, belonging to the brain regions in the left hemisphere in the Schaefer atlas, and **(F)** shows the second half, which represents the right hemisphere. This figure was created with MATLAB R2021a (www.mathworks.com).

When inspecting the model parameters optimized in the high-dimensional space, we found large fluctuations across different algorithm executions even for the same subject [**Fig 2, Supplementary Fig 4** in S1 Appendix]. Especially the optimized frequency parameters ${\left(f_{i}\right)}_{1\leq i\leq N}$ seem to cover the entire plausible range of values in [0.01, 0.1] Hz for the vast majority of brain regions i∈{1,…,N} [**Fig 2E,F,** **Supplementary Fig 4** in S1 Appendix]. Compared with this variance, the spread of the GoF values obtained in 30 algorithm executions appears to be low [**Fig 2D, Supplementary Fig 4** in S1 Appendix]. Indeed, for the considered example of the 103D parameter space with CMAES and the Schaefer atlas [**Fig 2, Supplementary Fig 4** in S1 Appendix], we found that the GoF values are narrowly distributed around the median 0.59 with interquartile range (IQR) 0.03, i.e., about 5% of the relative variability as given by the fraction IQR/median [**Fig 2D**]. On the other side, such a variability reaches on average 90% for the optimized frequencies [**Fig 2E**,**2F**], as well as 43% and 41% for the optimized parameters of coupling C and noise intensity σ, respectively [Fig 2B,2C]. Probably the largest variability is manifested by the optimized parameter of delay τ with IQR around 57.60 at zero median [**Fig 2A**]. The median value of zero however indicates that more than half of the performed algorithm executions for this subject yielded an optimal delay of τ = 0.

We verified the above findings for the entire subject cohort. Therefore, we evaluated the variability (IQR) of the approximated optimal model parameters and GoF values across algorithm executions for all subjects, see Supplementary Materials. We observed a persistently high variability of the model parameters for both atlases and optimization algorithms [**Supplementary Fig 5** in S1 Appendix]. Compared with the regional model parameters, i.e., frequencies ${\left(f_{i}\right)}_{1\leq i\leq N}$, the global parameters of delay τ, coupling C and noise intensity σ may show a slightly lower variability, but still a much higher one than that of the GoF values, see Supplementary Materials.

#### Correlations of optimized frequency distributions.

**Fig 3:**
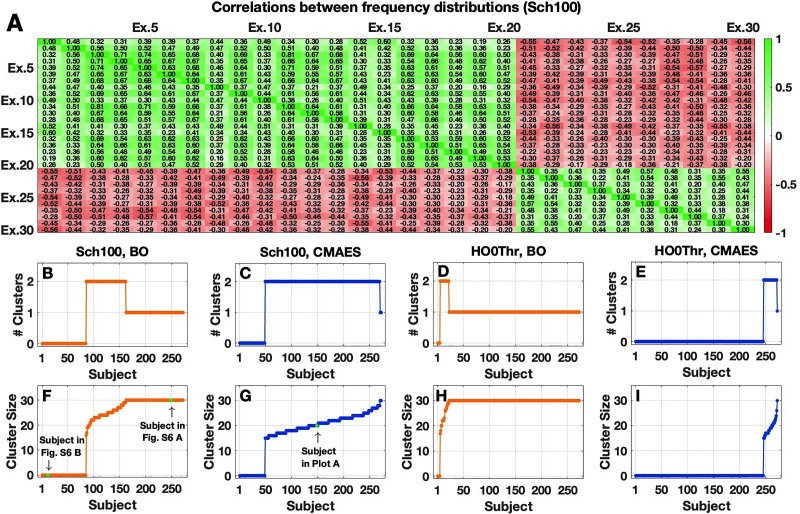
Structure of optimized model parameters in the high-dimensional cases. **(A)** Example of the correlations between optimized frequency parameter sets obtained at the model validation in the 103-dimensional parameter space via CMAES for the Schaefer atlas (Sch100). For a given subject, the correlations between any two sets of optimized frequency parameters ${\left(f_{i}\right)}_{1\leq i\leq N}$ obtained in different algorithm executions (Ex.) were calculated and then indicated in the table cells also highlighted in color. The algorithm executions were sorted, so that the cluster structure becomes observable. **(B-I)** Number of clusters and cluster size for all subjects and optimization approaches. **(B-E)** Subject-dependent number of the frequency clusters (# Clusters) for the atlases (Sch100 and HO0Thr) and methods (BO and CMAES) indicated in the titles. In each plot, the subjects were sorted according to the number of elements in the largest cluster as illustrated in the bottom plots. **(F-I)** Cluster size for all subjects, i.e., the number of algorithm executions in the largest frequency cluster. The number and size of the clusters were determined with help of the k-means clustering method. Both were set to zero if the absolute mean value of the off-diagonal elements in the correlation matrix (cf. **(A)**) was below 0.25 (corresponding to weak correlations between different frequency sets). The subjects considered in **(A)** and **Supplementary Fig 6** in S1 Appendix are highlighted in the plots **(F)** and **(G)**. This figure was created with MATLAB R2021a (www.mathworks.com).

We found that, despite the fluctuations, the values of the optimized frequency parameters are by no means entirely random across different algorithm executions. To illustrate this, we calculated the correlations between the values of the model parameters obtained for different optimization runs (with different initial conditions) for the same subject. More precisely, the N-dimensional vector ${\left(f_{i}\right)}_{1\leq i\leq N}^{q_{1}}$ of optimal frequencies obtained for one optimization run $q_{1}$ may show a pattern that tends to be strongly (anti)correlated with the optimal frequencies ${\left(f_{i}\right)}_{1\leq i\leq N}^{q_{2}}$ calculated for another optimization run $q_{2}$ for the same subject [**Supplementary Fig 4** in S1 Appendix, **Fig 3A**]. An example that visualizes such an (anti)correlation based on the optimized frequency parameter values is provided in **Supplementary Fig 4** in S1 Appendix. We observed *(anti)correlated clusters* of frequency patterns obtained for different optimization runs for individual subjects. All ($R_{\text{max}}$ = 30) runs can be split into two groups (clusters), where the vectors of the optimized frequencies strongly correlate with one another within the same cluster, but negatively correlate with those from the other cluster [**Fig 3A**]. The clusters may however vary in size for different subjects [**Fig 3B-3I**]. This means that one frequency pattern may be dominant for a given subject, and there is just one cluster of optimized frequency parameters, where all obtained frequency realizations strongly correlate with one another [**Supplementary Fig 6** in S1 Appendix]. For another subject, two anticorrelated frequency patterns (and respective clusters) can occur with almost equal or different probabilities [**Fig 3A**]. It may also happen that there are no clusters, and different optimization trials lead to weakly correlated frequency constellations [**Supplementary Fig 6** in S1 Appendix]. A PCA of the frequency patterns revealed that strong anticorrelations might be reflected by clear differences (separation) regarding the scores of the first principal component in the PC space [**Supplementary Fig 7** in S1 Appendix]. Weak correlations or the presence of just one dominating pattern in turn may align with increased variation in the second principal component but seem harder to be described by the PC scores [**Supplementary Fig 7** in S1 Appendix]. The fraction of explained variation by a given number of principal components differs considerably across subjects, while 13–16 components are on average needed to explain 80% of variation [**Supplementary Fig 7** in S1 Appendix]. This indicates that the space dimension of the optimized frequency parameters may be around 15 or higher. In general, the tendency to obtain two anticorrelated frequency patterns is more pronounced for the Schaefer atlas than for the Harvard-Oxford atlas, as more subjects exhibit two-cluster frequency realizations for the former parcellation than for the latter [**Fig 3B and 3I**]. Coming back to the analysis of the (GoF and parameter) variability across repeated algorithm executions for same subjects, we want to highlight that the IQR of GoF values was relatively low [**Supplementary Fig 5** in S1 Appendix] even when almost no clusters of frequency patterns could be observed across subjects [**Fig 3E**].

Nevertheless, the presence of anticorrelated frequency patterns, which reflect the discussed variability of the optimized model parameters, together with relatively small GoF changes can still be observed in cases where the high-dimensional optimization was informed by the results of the low-dimensional one [**Supplementary Fig 8** in S1 Appendix]. We confirmed the reported conclusions by taking into account the optimal parameter values approximated during the low-dimensional model fitting as starting points for the high-dimensional optimization problems (instead of random initial conditions that were considered above) [**Supplementary Fig 8** in S1 Appendix]. We also note here that the model includes a random noise term that can be an additional source of the observed variability at the optimization in the high-dimensional spaces.

Based on these findings, we suspect that the optimal solutions for a model validation in high-dimensional parameter spaces are located on a certain subspace of the domain of the goal function. We may refer to it as a *characteristic manifold of model fitting*, whose properties (such as a degeneracy) require a deeper investigation that builds on the methodological scope presented here.

### [II] Test-retest reliability of modeling results

In the following, we analyze how reliable the results of the model simulations are. We define the test-retest reliability of the modeling results as the ICC computed across repeated executions of the utilized optimization algorithms (see Methods). After investigating the reliability of the approximated optimal model parameters, we proceed to the corresponding sFC and GoF.

#### Reliability of model parameters.

**Fig 4:**
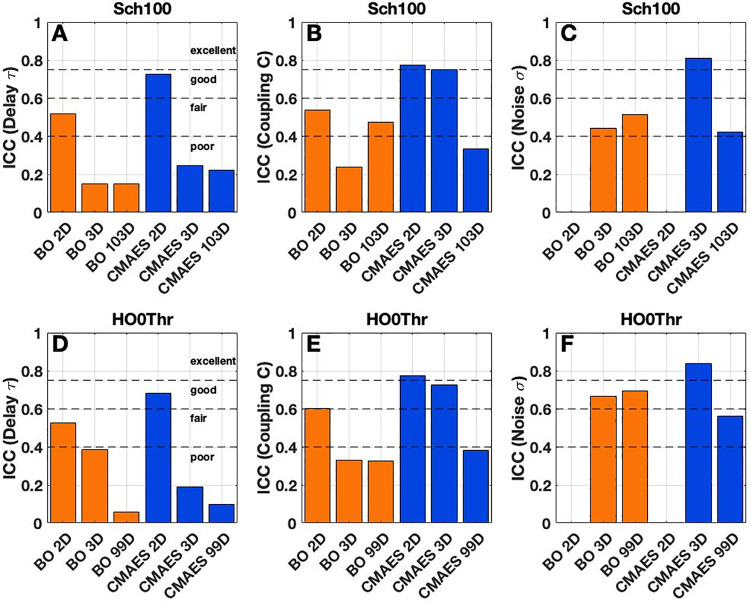
Reliability of the optimized model parameters of delay τ, coupling C and noise intensity σ. Colored bars illustrate the reliability of the modeling results as measured by the intraclass correlation coefficient (ICC). The considered optimization algorithms and parameter spaces are indicated on the horizontal axes along with the ICC scores on the vertical axes. Results are shown for **(A-C)** the Schaefer atlas (Sch100) and **(D-F)** the Harvard-Oxford atlas (HO0Thr). Dashed horizontal lines indicate the levels of *poor*, *fair*, *good* and *excellent* reliability in terms of the ICC as suggested in [72,73]. This figure was created with MATLAB R2021a (www.mathworks.com).

At first, we considered the optimized parameters of global delay τ, coupling C and noise intensity σ, and found that their reliability is influenced by the employed brain atlas and optimization method [**Fig 4**]. The reliability of parameter τ decreases for an increasing number of free model parameters. While it varies between *fair* (BO) and *good* (CMAES) for both atlases in 2D, it drops to a *poor* reliability for all other conditions that we tested [**Fig 4A**,**4D].** For the coupling parameter C, we also observed the highest values in 2D (BO: *fair*, CMAES: *excellent*), and subsequent drops for the other dimensions of the parameter space **[Fig 4B**,**4E**]. The only exception is given for BO and the Schaefer atlas, where the ICC reliability of C is smallest in the 3D case [**Fig 4B**]. The reliability of the noise intensity σ remains nearly unchanged for the transition from 3D to the high-dimensional cases for BO, staying within the *fair* (Schaefer atlas) or within the *good* range (Harvard-Oxford atlas) [**Fig 4C**,**4F**], which is in contrast with that of the delay and coupling parameters (but see **Fig 4B**). However, for CMAES, the reliability of the parameter σ clearly drops from the *excellent* to the *fair* range during the same transition to the high-dimensional spaces [**Fig 4C**,**4F**].

**Fig 5:**
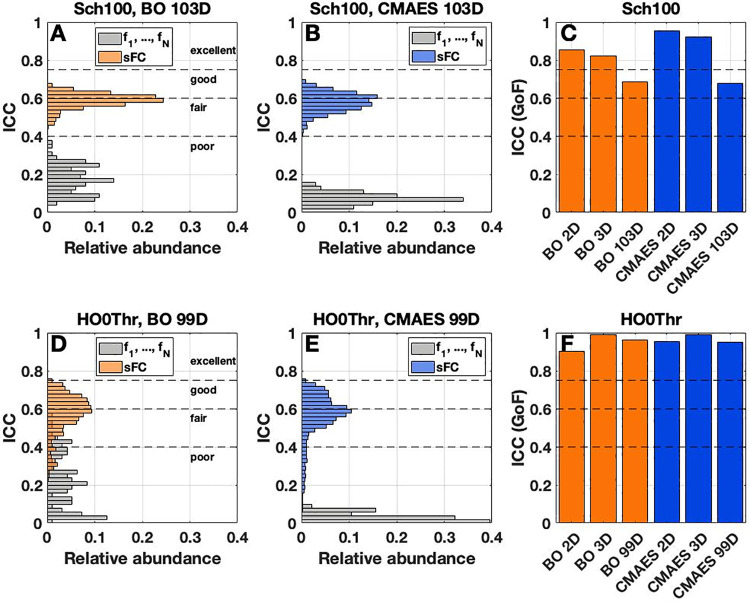
Reliability of the optimized frequency parameters and sFC matrix edges in the high-dimensional cases as well as of the GoF. **(A-B,D-E)** Colored, horizontal histograms visualize the distributions of the intraclass correlation coefficients (ICC) for the model parameters ${\left(f_{i}\right)}_{1\leq i\leq N}$ (gray) as well as for the above-diagonal edges of the matrices of sFC (orange and blue for BO and CMAES, respectively). The considered atlases, i.e., **(A-B)** the Schaefer atlas (Sch100) and **(D-E)** the Harvard-Oxford atlas (HO0Thr), are provided in the titles together with the utilized optimization algorithms and parameter spaces. **(C,F)** Colored bars illustrate the ICC scores of the GoF for all considered optimization algorithms and parameter spaces, which are indicated on the horizontal axes. The names of the atlases are again provided in the titles. Dashed horizontal lines indicate the levels of *poor*, *fair*, *good* and *excellent* ICC reliability as suggested in [72,73]. This figure was created with MATLAB R2021a (www.mathworks.com).

In the latter cases, we also evaluated the reliability of the frequency parameters ${\left(f_{i}\right)}_{1\leq i\leq N}$. We found that their ICC reliability hardly ever exceeds the threshold of 0.4 and can therefore be considered *poor* for all methods and parcellations [**Fig 5A**,**5B**,**5D**,**5E**, gray histograms].

In summary, the reliability of the three model parameters of delay τ, coupling C and noise intensity σ shows a rather complex behavior as measured by the ICC. We can, however, conclude that the reliability of the parameters τ, C and σ becomes lower when the number of free model parameters increases for CMAES. For BO, this is also the case, except for C (Schaefer atlas) and σ (both atlases). In the high-dimensional cases, the optimal delay, coupling and frequency parameters mainly demonstrate a *poor* reliability. Nevertheless, the optimal noise intensity can still be obtained with *fair* or even *good* (BO, Harvard-Oxford atlas) reliability, which indicates that its variability within subjects can be lower than between subjects for multiple executions of the optimization methods.

#### Reliability of sFC and GoF.

In this step of our analyses, we investigated the ICC reliability of the optimal sFC matrices generated in each algorithm execution. Here, the reliability of every individual matrix element above the diagonal was calculated across subjects (see Methods), and the distributions of the obtained values were inspected. We started with the high-dimensional cases, where it turned out that the sFC matrices feature reliable edges ranging mainly between a *fair* and *good* quality [**Fig 5A**,**5B**,**5D**,**5E**, orange and blue histograms]. These matrices can nevertheless be outperformed by their equivalents from the 2D and 3D cases which, for their part, may even reach an *excellent* reliability, except for BO with the Schaefer atlas [**Supplementary Fig 9** in S1 Appendix].

We further evaluated the ICC reliability of the GoF values obtained for repeated runs of the optimization procedures. Here, we observed a decreasing trend for both algorithms and the Schaefer atlas, with the highest reliability in the case of the two-dimensional parameter space (*excellent*), followed by 3D (*excellent*) and 103D (*good*) [**Fig 5C**]. For the Harvard-Oxford parcellation, the GoF values reached an *excellent* reliability in all considered cases, including the high-dimensional ones [**Fig 5F**].

In summary, we observed an sFC reliability of *good* quality together with high ICC values for the GoF (*good* and *excellent* reliability) even for the high-dimensional model fitting. These findings are in alignment with the low GoF variability for single subjects described above but needed to be verified given the remarkable fact that the optimized model parameters, which generate such sFCs and GoF values, tend towards a *poor* reliability [**Figs 4**,**5**].

To deeper investigate the two modeling results (sFC, GoF) that turned out as the most reliable ones across optimization runs, we used them to compute the subject specificity, see Supplementary Materials. The specificity characterizes the similarity/discrepancy between intra- and inter-subject properties like average FC correlations within subjects vs. average FC correlations between subjects, for instance. We tested ES- and difference-based approaches to compute the specificity and analyzed the impact of the dimension of the parameter space.

We found that the specificity indices frequently remained within narrow ranges of values, and we did not find a clear trend that would indicate an enhancement or reduction of the specificity depending on the dimension of the model parameter space considered for the model validation [**Supplementary Figs 10,11,12,13** in S1 Appendix].

At this point, we emphasize that the model validation in high-dimensional parameter spaces much better approximates the measured brain activity as the simulated data (sFC) closely replicate the empirical ones (eFC). A comparably low correspondence between empirical and simulated data in the low-dimensional parameter spaces can be balanced by an enhanced reliability of the model parameters and connectivity patterns. While the reliability of sFC edges and GoF values can reach the fair, good and excellent ranges also for the model validation in the high-dimensional parameter spaces, there remains the high variability of the approximated optimal model parameters. This may play a role in further applications, where it may often be desirable to extract *one* final outcome per subject. However, as we will see in the next part, sampling from parameter distributions or selecting one solution based on given characteristics may open up new possibilities for further successful usage of the simulated data. Our results for the high-dimensional cases do bear a potential for a better detection of inter-individual differences despite the observed variability of model parameters.

### [III] Application of modeling results to phenotypical data

In this section, we focus on the potential benefit of the whole-brain model simulations for the detection of phenotypical differences in the data. Therefore, we split our cohort of subjects into males and females and analyze the modeling results separately for both groups (see Methods).

#### Sex differences in GoF distributions.

**Fig 6:**
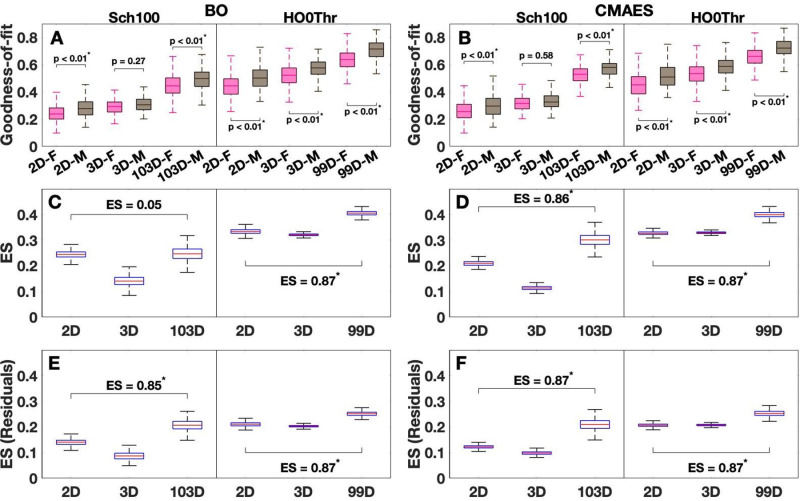
GoF values for males and females together with the corresponding effect sizes (ES) of the observed differences. (A-B) Colored boxplots show the distributions of the median model fits obtained for males (brown) and females (pink) in 30 algorithm executions with random initial data (option 2, see Methods). The considered dimensions of the parameter spaces combined with the group abbreviations of males (M) or females (F) are indicated on the horizontal axes along with the detected GoF values on the vertical axes. Solid vertical lines separate the results for the Schaefer atlas (Sch100) and the Harvard-Oxford atlas (HO0Thr). The names of the utilized optimization algorithms are given in the titles. Statistically significant sex differences as assessed by the Wilcoxon rank-sum test are marked with an asterisk. The significance level of 5%, p < 0.05, has been Bonferroni-corrected for multiple comparisons. The corrected p-values of the sex differences are indicated in the plots. (C-D) Boxplots illustrate the distributions of the ES for the sex differences observed during a random selection of one of the 30 available algorithm executions for every subject and a subsequent comparison of the corresponding GoF values across both groups (1000 repetitions). The ES of the changes in ES from the low- (2D) to the high-dimensional (99D, 103D) cases are indicated in the plots. The Wilcoxon rank-sum test was applied. Statistically significant differences are marked with an asterisk. The significance level of 5%, p < 0.05, has been Bonferroni-corrected for multiple comparisons. (E-F) Same as (C-D), but the ES of the sex differences were computed for the GoF residuals obtained by regressing out the intracranial volume of the individual subjects from the GoF values. This figure was created with MATLAB R2021a (www.mathworks.com).

We observed significantly higher GoF values for males during the model fitting procedure, with a regular exception given by the 3D cases with the Schaefer atlas [**Fig 6A**,6B]. Among the optimization algorithms, we obtained mostly consistent results, where the sex-related GoF differences are significantly stronger for the high-dimensional model fitting than for the low-dimensional cases (except for BO with the Schaefer atlas) [**Fig 6C**,6D]. To be more precise, the median ES of the differences between males and females for the Schaefer atlas ranges around 0.21 in the 2D case and climbs to roughly 0.3 for CMAES (ES = 0.86 between 2D and 103D) in the high-dimensional model fitting [**Fig 6D**], but not for BO, where it remains at the approximate level it had in 2D [**Fig 6C**]. The situation is different for the Harvard-Oxford atlas, where the ES of the sex differences are in general higher (around 0.33 in both 2D and 3D [**Fig 6C**,6D]) than their counterparts for the Schaefer atlas. An obvious increase of the GoF differences between males and females was observed in the high-dimensional case [**Fig 6C**,6D]. It approaches a moderate ES around 0.4 for both algorithms (ES = 0.87 between 2D and 99D). So, except for BO with the Schaefer atlas, the high-dimensional model validations seem to strongly enhance the observed differences between males and females in GoF values (with very strong ES of the differences between the low- and high-dimensional cases [**Fig 6C**,6D]).

Subsequently, we investigated whether the sex differences in GoF could be related to the differences in intracranial brain volume between males and females. We therefore applied linear regression to regress out the intracranial volume from the GoF values and repeated the analysis from above for the respective GoF residuals (see Methods). Here, we indeed observed a reduction of the differences between males and females, which is indicative of an impact of the intracranial brain volume on sex differences in GoF values [compare **Fig 6C**,6D with **Fig 6E**,6F]. Nevertheless, we also confirmed that the sex differences in the modeling results are more pronounced when the model validation is performed in high-dimensional parameter spaces [**Fig 6E**,6F]. Even for BO with the Schaefer atlas, which previously did not confirm the trend, we observed a very strong increase of the differences between males and females when comparing the low- and high-dimensional cases (ES = 0.85 between 2D and 103D) [**Fig 6E**].

#### Sex differences in empirical data, model parameters and FC strength.

To assess the actual benefit of the data generated by whole-brain model simulations, we also searched for similar patterns in empirical data. We therefore considered the structure-function relationship as given by the correlation between SC and eFC. It appeared as the most straightforward empirical alternative to the GoF of the modeling results. Here, we found larger correlation values for the females than for the males for both atlases [**Supplementary Fig 14** in S1 Appendix], in contrast to the GoF values, see also [81]. However, the absolute ES of the sex differences did not exceed 0.2 (-0.13 for the Schaefer atlas and -0.19 for the Harvard-Oxford atlas), which was regularly surpassed in the modeling outcomes [**Fig 6C**,6D]. This result provides further evidence of the practical utility of whole-brain model simulations, which can, as in the considered case, reveal differences between males and females that are hardly observable in empirical brain imaging data.

We note that our data do not yet show equipollent differences between males and females when the analysis was based on the optimized values of the model parameters instead of the GoF values. The ES remained very low in most cases. Significant and relatively strong increases in the (still low) ES could only be observed during the transition from low- to high-dimensional parameter spaces for the coupling parameter C for both atlases and optimization methods and slight increases for the noise intensity σ for the Schaefer atlas and both methods [**Supplementary Fig 15** in S1 Appendix]. For the other cases and model parameters, the sex differences can even decrease for the high-dimensional model fitting [**Supplementary Figs 15,16** in S1 Appendix]. Especially the frequency parameters ${\left(f_{i}\right)}_{1\leq i\leq N}$ did not seem to contain information suitable for differentiating between subjects [**Supplementary Fig 16** in S1 Appendix]. By contrast, the sex differences in the GoF values can frequently and strongly increase for the high-dimensional model fitting, where the ES may reach up to 0.4 in the median [**Fig 6**].

**Fig 7:**
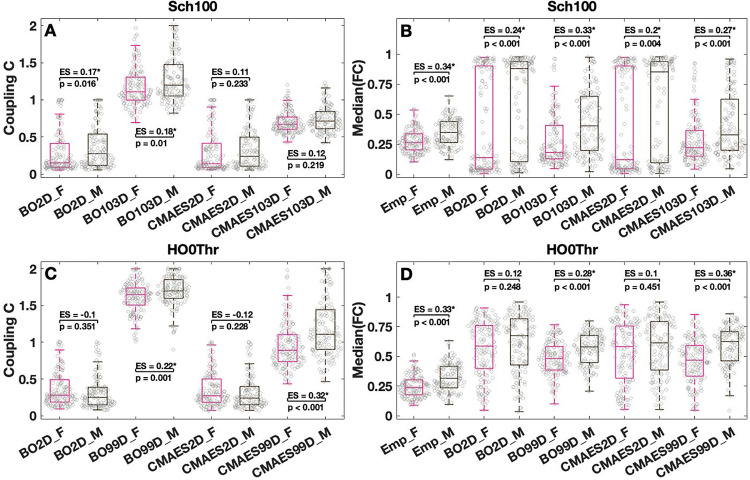
Distributions of optimized coupling parameters and median FC strengths for males and females. Colored boxplots together with gray circles in the background show the distributions of **(A,C)** the values of approximated optimal coupling parameters C and **(B,D)** the median connectivity strength in the FC matrices for males (brown) and females (pink). Empirical (eFC, Emp) as well as simulated (sFC) data were considered. For every subject, the eFC from the concatenated sessions as well as the sFC matrix and coupling parameters yielding the highest GoF across algorithm executions were selected (option 1, see Methods). We refrained from including the 3D cases in the figures as we found no remarkable differences to the 2D cases. The names of the utilized optimization algorithms and considered dimensions of the parameter spaces combined with the group abbreviations of males (M) or females (F) are indicated on the horizontal axes along with the median FC and optimal *C* values on the vertical axes. The names of the considered atlases are given in the titles, i.e., **(A,B)** the Schaefer atlas (Sch100) and **(C,D)** the Harvard-Oxford atlas (HO0Thr). Statistically significant sex differences as assessed by the Wilcoxon rank-sum test are marked with an asterisk. The significance level of 5%, p < 0.05, has been Bonferroni-corrected for multiple comparisons. The effect sizes (ES) and corrected p-values of the sex differences are indicated in the plots. This figure was created with MATLAB R2021a (www.mathworks.com).

Nevertheless, as the approximated optimal parameters constitute one major source of new, model-based information regarding the brain dynamics of a given subject, we investigated the practical utility of the model parameters further. We attempted to suggest a justified strategy of selecting one of the 30 fluctuating solutions per subject and find relations to other properties of the neuroimaging and behavioral data. In this endeavor, we could repeatedly detect slightly higher coupling values for males in the high-dimensional cases [**Fig 7A**,7C] when selecting one of the available 30 solutions per subject based on the following three options: Choose the algorithm solution from the execution yielding (1) the highest GoF, (2) the median GoF or (3) the minimal ${\left\|\text{sFC}-\text{eFC}\right\|}_{\text{F}}$, see also [**Supplementary Figs 17,18** in S1 Appendix]. We controlled our results for GoF outliers.

Trying to relate the pattern of higher coupling parameter values for males to the practical role of the coupling parameter in the considered whole-brain model, we inspected whether there are also sex differences in the FC strength. We therefore compared the respective median FC values when we selected one FC matrix per subject from the three mentioned options. Here, we indeed found that the connectivity seems to be significantly stronger for males than females despite a wide overlap [**Fig 7BD**,]. For the eFC, we observed a significant ES of 0.34 and 0.33 for the Schaefer and Harvard-Oxford atlas, respectively. While the ES of sFC in the low-dimensional cases are clearly lower (0.1 to 0.24 for option 1) across all tested scenarios, they approach or even exceed (ES = 0.36 for CMAES with HO0Thr, option 1) the empirical values in the high-dimensional cases. Very similar trends can be identified for option 2 [**Supplementary Fig 17** in S1 Appendix] and 3 [**Supplementary Fig 18** in S1 Appendix]. So, higher values for the optimized model parameter of coupling C in the high-dimensional cases seem to align with higher values of the median connectivity strength in the FC matrices, which may explain the observed stronger FC in males also in empirical data.

#### Model-based sex classification.

**Fig 8:**
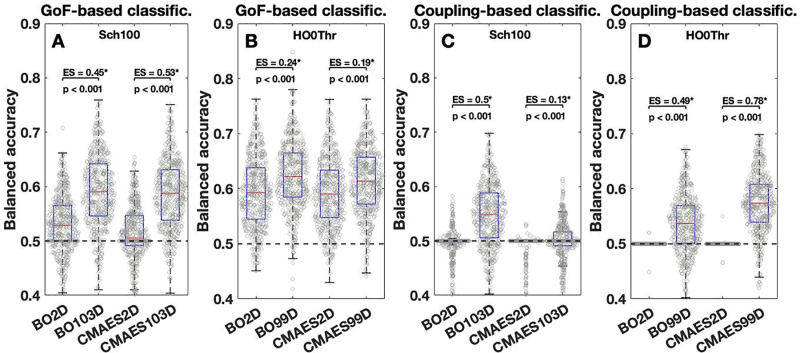
Model-based sex classification accuracies. **(A-D)** Boxplots together with gray circles in the background visualize the balanced accuracies of a logistic regressor trained for sex classification based on **(A-B)** the GoF or **(C-D)** the optimized values of the coupling parameter C. For every subject, the GoF and parameter values derived from the algorithm execution yielding the highest GoF were selected (option 1, see Methods). We refrained from including the 3D cases in the figures as we found no remarkable differences to the 2D cases. The names of the utilized parameter optimization algorithms and parameter spaces are provided on the horizontal axes along with the balanced accuracy values on the vertical axes. Dashed horizontal lines indicate the chance level of 50%. Indications of which property (GoF or coupling parameter) was used for classification (classific.) are provided in the titles. The names of the considered atlases (Sch100 for the Schaefer atlas and HO0Thr for the Harvard-Oxford atlas) are given in the plots. Statistically significant performance gains between low- and high-dimensional parameter spaces as assessed by the Wilcoxon rank-sum test are marked with an asterisk. The significance level of 5%, p < 0.05, has been Bonferroni-corrected for multiple comparisons. The effect sizes (ES) and corrected p-values of the increases are indicated in the plots. This figure was created with MATLAB R2021a (www.mathworks.com).

Finally, we inspected the applicability of the modeling outcomes to a sex classification task as an example of the investigation of inter-individual variability. We trained the machine learning classifier, a logistic regressor, separately on the GoF values as well as on the optimized model parameter values and inspected the performance changes between low- (2D) and high-dimensional (99D, 103D) parameter spaces. (The 3D cases did not show outstanding differences to the 2D cases.) It turned out that the GoF and optimized coupling parameter values are useful features for classification in the high-dimensional cases, while the remaining parameters are not. We found balanced prediction accuracies centered mostly around the chance level of 50% for the model parameters of delay τ, noise intensity σ and frequencies ${\left(f_{i}\right)}_{1\leq i\leq N}$ (not shown), which resonated with a poor practical utility of these quantities. For the features of GoF and coupling C, we however observed median accuracies reaching up to 62% and 57% in the high-dimensional cases, respectively, when the GoF or coupling parameter value obtained from the algorithm execution yielding the highest GoF (option 1) were selected [**Fig 8**]. The ES between low- and high-dimensional scenarios can reach 0.53 and 0.78, respectively [**Fig 8**]. We also tested the classification performances for the options 2 and 3 of solution selections [**Supplementary Figs 19,20** in S1 Appendix] and found that option 1 might exhibit the most consistent and pronounced classification improvements for the high-dimensional cases, especially for the coupling parameter C. Option 1 therefore appears to be most suited for the applications presented here.

We conclude that the validation of the whole-brain models in high-dimensional parameter spaces is beneficial for studying differences between males and females in simulated resting-state data. For selected modeling quantities, the extent of observable sex differences exceeded those that are present in purely empirical data. We found in particular that the gap in the respective GoF value distributions between male and female subject groups can be expanded with a very strong ES of about 0.87 when the model validation is performed in high-dimensional parameter spaces. This conclusion still holds after regressing out the intracranial brain volume of the individual subjects. Additionally, we found significantly higher classification accuracies for a sex classifier trained on the modeling output from the high-dimensional cases, where one out of 30 available algorithm solutions was selected as the final output for a given subject. Our results indicate beneficial effects of the model validation in high-dimensional parameter spaces and a general importance of involving modeling results in the analyses and interpretations of brain and behavior.

## Discussion

In this work, we demonstrated that selected mathematical optimization algorithms allow for precise model simulations of resting-state brain activity. Remarkably, the validation of whole-brain models featuring high-dimensional model parameter spaces can be performed even for large subject cohorts. We showed that the transition from low- to high-dimensional parameter spaces improved the observed model fitting quality (GoF) dramatically [**Fig 1**]. This enhancement was accompanied by an increased intra-subject variability of optimized model parameters [**Fig 2**], which constitutes an issue that requires further attention. We however found that despite the fluctuating parameters, the obtained GoF and also sFC remained relatively stable and reliable [**Fig 5**]. Finally, we could show that the sex differences in simulated data became much more pronounced when the fitting of the whole-brain models to empirical data was performed in the high-dimensional parameter spaces. The resting-state brain dynamics of males could be better replicated by the employed model than those of females [**Fig 6**], which was particularly evident in the high-dimensional cases, where we observed significantly higher classification accuracies compared to the low-dimensional cases [**Fig 8**]. Altogether, this highlighted the practical utility of the high-dimensional model fitting. It depicted the used optimization approach and dynamical model as useful complements to empirical data when dealing with phenotypical differences and brain-behavior relationships.

We want to stress an important finding, which pertains to the location of approximated optimal model parameters. We observed that both algorithms frequently converged to more than one optimal solution when the high-dimensional cases were considered, while the GoF and sFC often varied only slightly. This let us speculate about the presence of a manifold of optimal model parameters in the high-dimensional cases. We cannot yet provide a mathematical proof of the existence of a manifold, which is far from trivial. However, such a notion was supported by the fact that the reliability (as measured by the ICC across repeated algorithm executions) of the GoF as well as of the edges in the sFC matrices ranged mainly between *good* and *excellent* despite a *poor* to *fair* reliability for the majority of model parameters in the high-dimensional optimizations [**Figs 4**,**5**]. The two mentioned quantities (GoF and sFC) therefore appear much more reliable than the model parameters that generate them and can thus be considered as modeling outcomes qualified for further employments of model application (studying phenotypical differences, for instance). The low, but still present intra-subject variability of GoF and sFC may be related to the interplay of stochastic factors such as the noise term in the considered whole-brain model and the random numbers generated and processed by the algorithms during their iterative optimization procedures.

We infer that it is indeed possible and also favorable to perform the model validation in high-dimensional model parameter spaces by employing relatively simple mathematical optimization algorithms. As an initial way to cope with the observed variability of the approximated optimal model parameters, we proposed to either sample (GoF and parameter) values from the solution distributions obtained by repeated optimization runs or to select one final solution based on the highest GoF or other options, for example. The insights deduced from the optimized model parameters may, admittedly, still require deeper investigations, but the obtained GoF and sFC already provided solid results for the replicability of human brain dynamics and differentiability of individual phenotypical properties.

### Computational resources

We stress that a considerable (but still tractable) amount of computational resources is necessary to perform the model fitting in the high-dimensional parameter spaces for both algorithms [**Supplementary Fig 3** in S1 Appendix]. This might limit the applicability of such an optimization approach in case of low-budget computational resources. Nevertheless, both considered optimization methods outperformed the grid search parameter optimization starting from the 3D cases in this respect [42]. While more computational time is required for the high-dimensional cases as compared to the low-dimensional ones, the amount necessary to optimize in a 99D or 103D parameter space is still comparable to that of a 3D grid search [**Supplementary Fig 3** in S1 Appendix], not to say about more dimensions. In view of the growing technological progress and current practices to work with *big data*, however, we are convinced that more computational resources will be dedicated to the brain research and modeling. This means that the discussed computation time may be no longer a great issue in the nearest future. Combining the presented gains in phenotypical differences with the much-improved quality of the model validation, we conclude that the invested computational costs are justified.

### Selection of whole-brain model, atlases and optimization algorithms

We worked with the Kuramoto model of coupled phase oscillators, which is one of the simplest models suited for simulating the features of resting-state brain activity [83]. Given our intention to study the effect of a drastic increase in the number of optimized model parameters (and herewith the complexity of the model), it appeared reasonable to select this basic modeling approach for our study. In addition, its simple structure made it easier to investigate the immediate impact of the number of free parameters on the simulated dynamics. In line with numerous studies [54,55,84] that praised the Kuramoto model for providing a balanced tradeoff between complexity and plausibility in whole-brain modeling, we therefore decided to work with this model. We do not suspect that our qualitative results (e.g., increase in GoF for high-dimensional cases) depend on the selected modeling approach. However, we cannot provide any statement on whether or not the pattern of anticorrelated parameter constellations would generalize to other model validations in high-dimensional parameter spaces, especially for other regional model parameters than the considered natural oscillation frequencies. That should be tested in future studies. The GoF differences between males and females, for their part, were also observed for a network model of limit-cycle oscillators in a related work [81], which supports our expectations that the results reported in this study for the phenotypical data can be generalized to other models.

We considered two paradigmatically distinct brain atlases. They define how the brain is parceled into distinct brain regions that are treated as network nodes in the model. While the Schaefer atlas [45] is based on functional MRI data, the Harvard-Oxford atlas [46] is inspired by the human brain anatomy, i.e., the presence of sulci and gyri on the cortical surface. Our motivation was to study the modeling results for a sample of both the functional and the anatomical parcellation approach with comparable numbers of brain regions, similarly as in [44]. Related works [8,85] deal with systematic atlas variations and the resulting impacts on the whole-brain modeling. An important extension of our work might encompass the comparison of coarser and finer parcellations from the same paradigmatic approach and the impact on the obtained GoF, for instance. The cited works have shown that the quality of the model fit does not depend on the selected paradigmatic approach alone. Indeed, it was reported that the anatomical Harvard-Oxford atlas outperformed the functional Schaefer parcellation in terms of GoF for many applications of FC simulation. Interestingly, in our study, this was also observed in the high-dimensional cases, where the model for HO0Thr featured 99 parameters and therefore 4 less than the one for Sch100 (103 parameters). Conversely, it has been shown that the (functional) Shen atlas [86] may yield a very high GoF as compared to Sch100 [8,85]. While we cannot provide a clear answer on what causes a particular atlas to perform better or worse than others, we know that selected properties of empirical data variables such as the empirical structure-function relationship strongly correlate with the obtained GoF [8]. Differences in the values of these variables for different parcellations may align with the observed differences in GoF.

The selection of the utilized mathematical optimization algorithms was based on our previous work [42], where we thoroughly discussed the advantages and drawbacks of several approaches for model validation. BO and CMAES emerged as the best methods in the tested low-dimensional parameter spaces and therefore had the greatest potential for the high-dimensional cases. While CMAES, the population-based algorithm, generated the most stable results with respect to the spread of solutions, BO, the global probabilistic approach, could convince potential users with its most efficient use of computational resources. Having applied both methods for the high-dimensional model validation in this study, we can conclude that they are indeed applicable for such kinds of optimization problems. We however note that, in terms of the rise in the invested resources [**Supplementary Fig 3** in S1 Appendix], the transition to the high-dimensional cases seemed to be a smaller problem for CMAES than for BO, which is deemed most successful in optimization problems featuring up to 20 free variables [63]. Also, CMAES seemed to give quite a better fit for the Schaefer atlas in the high-dimensional cases than BO [**Fig 1**]. Therefore, together with a recommendation for CMAES, we note here that another exhaustive comparison of both algorithms might be necessary, which was not the main intention of this study though. The observed results for the parameter variability, reliability and, also, application to phenotypical data did not evoke any solid impression that one of the applied methods is clearly better or worse than the other one.

In this study, we assessed the quality of the model validation with help of the well-established and commonly used Pearson correlation coefficient between static sFC and eFC, see also recent works [87–89]. One may also aim to apply the algorithms to newer, more elaborate measures that include dynamic FC (dFC), for instance.

### Optimal manifolds and concerns about overfitting and overparameterization

Observing several parameter constellations that all lead to comparable values of the goal function in a mathematical model is a known issue. Indeed, the so-called problem of parameter identifiability in dynamical systems has been known for a long time already [90–96]. In general, a distinction can be made between structural and practical identifiability [97]. While the former pertains to the more fundamental impossibility of estimating unique optimal model parameters under perfect experimental conditions (continuous and noise-free data), the latter applies to all cases, where the condition of a perfect experimental setting cannot be fulfilled because of noise-related inaccuracies, for instance. Several approaches to tackle identifiability problems have been suggested in the literature [98–101]. Typically, they are applied to reduce the number of model parameters based on their mutual interdependencies and/or the information extracted from the Fisher Information Matrix [35,36]. In view of our results, we can suppose that the positively correlated frequency patterns yielding similar GoF values and sFC matrices may be related to a form of practical identifiability: Across repetitions, the algorithms may produce several approximations of the same optimal point, which are variable in their quality (distance to the actual optimum). The observed anticorrelations between the solutions of repeated algorithm executions for the same subject may however go beyond the standard parameter identifiability issue. Besides the matter of a potential degeneracy [75,102,103], previous studies have reported a so-called *neural redundancy* in the brain, meaning that different patterns of neural activity generate the same outcome [104,105]. It is indeed possible that the algorithm solutions constitute distinct samples of optimal points which are all located on a high-dimensional manifold. In that sense, the anticorrelation between two solutions may indicate that such two points are found on opposite or reflected (mirrored) locations. Both of them still generate the same (or very similar) outcomes in terms of simulated resting-state FC and quality of the model validation. Our work thus also contributes to the existing literature by providing useful insights which are in alignment with the idea of neural redundancy in the brain. Optimal model parameters being located on a subject-specific manifold in the parameter space might characterize an individual subject’s personal profile.

Further, we note that the considered model parameters, e.g., the frequencies of the oscillators, are motivated by considering the brain as a network of active units (regions). A possible reduction of the parameter space dimension by an irreversible parameter change, redefinition and compression whatsoever would inevitably cause challenges of result interpretability. Based on that, we argue that overparameterization is not much of an issue in the scenarios we analyzed. The sets of model parameters that appear to be natural for the brain dynamics should be analyzed as they are if the interpretability of the derived conclusions is important.

A similar reasoning applies to the question of overfitting. Kong and colleagues [28] reported a significantly worse fit when 205 model parameters optimized in a training set were applied to a test set for a mean-field model based on group-averaged SC and FC data. The authors therefore advocated for a constraint of high-dimensional model parameter spaces as the model parameters should not be too flexible and free. They proposed to parameterize local circuit properties with help of both anatomical and functional gradients. In our study, we were working with personalized models that were derived from and validated against neuroimaging data of individual subjects. There were no models for an entire subject group here, which could be overfitted to the data of this given group and then would fail if applied to another group. In our approach, we are interested in the best possible model for every single subject individually, which can be considered a digital twin for brain investigation *in silico* [23]. We however recognize that the high-dimensional parameter optimization considered in this study may pose a limitation due to a potential risk of overfitting when the approximated optimal model parameters were to be used for fitting whole-brain models for other (groups of) subjects. While the still relatively moderate fraction of variance explained by the model [**Fig 1**] may not immediately indicate such a problem, additional investigations might be necessary in this respect.

At this point, we stress that the variability in optimized parameters observed across repeated algorithm executions for same subjects does not yet allow for an unambiguous selection of one specific solution that shall be used for further applications. Our reflections on the clustering of different frequency patterns can be seen as a first step to group together solutions with equivalent properties. Additionally, we offered three simple options on how to select one from the available algorithm executions as the final outcome for a given subject. We may advocate for choosing the solution yielding the highest GoF for subsequent analyses as it provided the best results in our applications. All results were however obtained during an optimization procedure which aimed at the maximization of the Pearson correlation between sFC and eFC (i.e., GoF) only. As we have seen, this condition leaves the parameters a certain amount of freedom since they “only” have to be located somewhere on the presumed high-dimensional manifold of optimal parameters. We suspect that an inspection of the results with focus on other features of interest such as graph-theoretical network properties in combination with norm-based distances to the empirical data (cf. option 3) may help in further reducing the variability in the solutions that can be treated as distributions (comparable in their mean, median etc.) for now, as in related studies [23,34]. Another approach would be to intensify the work with a PCA of the optimized model parameters. This might provide another way to estimate the dimensionality of the parameter space occupied by the parameters and select leading variables of the parameter distributions.

### Phenotypical differences

The observed sex differences in model fitting quality (GoF), which persist even after the regression of the intracranial volume, constitute an important finding of this study. With one exception given by BO for the Schaefer atlas, we have observed very strong and significant increases in the ES between GoF distributions for males and females when transitioning from the model validation in the two-dimensional to the high-dimensional parameter spaces [**Fig 6**]. This implies that the model-based connectome relationship of the function-to-function fitting (sFC to eFC) may be helpful in distinguishing between males and females in brain research. The GoF of dynamical whole-brain models provides a measure that quantifies how easy/difficult it is under the given modeling conditions to mimic the resting-state brain dynamics of a given human being. Pronounced differences in this quantity (or others, like the model parameters) provide supplementing information going beyond that encoded in purely empirical data and can thus enhance the differentiability between subjects. Especially the models optimized in high-dimensional parameter spaces emerge as useful complements to empirical observations. Our improved classification results underline this claim.

In fact, the extraction of well-pronounced differences between males and females from functional brain imaging data has been far from trivial for a long time. Many studies reported no or only very small and inconsistent findings especially when dealing with functional data related to cognitive tasks in psychological domains [106–109]. But also for resting-state fMRI data, it has been claimed that no differences between males and females can be detected [110]. At roughly the same time, however, other researchers published results which indeed indicated the presence of sex differences in certain resting-state networks and other elements of the functional brain organization [111–114]. In the course of time, the technological progress led to deeper insights into this topic. On the one hand, more complex methods such as machine learning approaches for classification could be applied and, on the other hand, studies over longer time periods as well as studies including larger subject cohorts could be conducted [79,115–120]. Our rationale for applying the modeling results to sex differences was based on one of these latter works, where intriguing classification results based on resting-state FC were obtained, despite the absence of a clear-cut dimorphism between males and females in the functional data [118]. It has been argued that, with the availability of new methodological approaches such as statistical learning, the general question should not be whether or not differences between males and females exist in the brain, but rather how large and meaningful they are compared to the overall intra- and inter-individual brain variability of subjects [121].

We regard our findings as an important contribution to this topic. The model validation in high-dimensional parameter spaces opens new perspectives for more personalized models that can reflect additional subject-related properties. In particular, we observed that the replication of resting-state FC by model simulations appeared to be easier (larger GoF) for the whole-brain model when the data of male participants were utilized as compared to those of females. This trend was mostly pronounced in the high-dimensional cases, regardless of which of two inspected optimization algorithms and brain atlases were considered. So, while our fitted results approached the empirical data more and more closely from one side, we were also able to outperform the empirical data from another side, here by revealing increased differences between males and females (which, if desired, could be mitigated by considering males and females separately throughout the entire modeling approach or by applying linear regression on the sex-related GoF values prior to further applications, for example). Further, we stress that our applied regressor was relatively simple and reached a classification accuracy of 62% based on one scalar value (GoF or optimal coupling strength, i.e., always one feature regardless of the dimension of the parameter space) only. This accuracy is not very high, and multivariate analyses on empirical data might likely outperform our results. However, analyses combining both empirical and simulated data may prove superior to approaches that resort to only one of the mentioned data types. In this way, empirical approaches might benefit from considering the simulated data obtained at the model fitting in the high-dimensional parameter spaces as additional features. Especially the conflation of several features that we treated in isolation so far may bear a huge potential. We therefore suppose that the personalized validation of mathematical whole-brain models in high-dimensional parameter spaces can potentially contribute to further explorations in the field of phenotypical differences in the brain as well as other research topics focusing on the link between brain and behavior. In the considered example of sex differences, our results confirmed and extended the insights extractable from empirical data, which might be favorable for the long-term idea of individualized care and precision medicine [122,123].

## Conclusions

We have shown that practical and efficient tools for model validation in high-dimensional parameter spaces are available nowadays. Their computational feasibility and economy rendered them suitable for applications even on large subject cohorts. The transition from the low- to the high-dimensional parameter spaces was marked by significant increases in the fitting quality of the whole-brain models. In the best case, the GoF could even double. The reliability of sFC and GoF remained high even after a high-dimensional model validation, which suggests the usage of these quantities for further applications. We also observed that differences in GoF values and optimized model parameters of coupling strength were more pronounced between males and females in the high-dimensional cases and therefore led to significantly higher sex classification accuracies. Our study can contribute to an improved, personalized modeling of human brain dynamics, which could be helpful for a deeper understanding of the latter. Whole-brain models validated in high-dimensional parameter spaces may play a supporting role in personalized medical decision making as they may be used as a risk-free test bench for clinical research and application.

## Supporting information

S1 Appendix Supplementary Materials. (DOCX)
