# Supplementary material for: Exploring dynamical whole-brain models in high-dimensional parameter spaces
Source: PLoS One. 2025 May 12;20(5):e0322983. doi: 10.1371/journal.pone.0322983 (PMC12068738; doi:10.1371/journal.pone.0322983)
Supplement: S1 Appendix — (DOCX) [file pone.0322983.s001.docx]

**Supplementary Materials**

**Exploring dynamical whole-brain models in high-dimensional parameter spaces**

*Kevin J. Wischnewski* ^1,2,3^*, Florian Jarre* ^3^*, Simon B. Eickhoff* ^1,2^ *& Oleksandr V. Popovych* ^1,2,*^

^1^ Institute of Neuroscience and Medicine – Brain and Behaviour (INM-7), Forschungszentrum Jülich, Germany

^2^ Institute of Systems Neuroscience, Medical Faculty and University Hospital Düsseldorf, Heinrich Heine University Düsseldorf, Germany

^3^ Institute of Mathematics, Faculty of Mathematics and Natural Sciences, Heinrich Heine University Düsseldorf, Germany

^*^Corresponding author ([o.popovych@fz-juelich.de](mailto:o.popovych@fz-juelich.de))

| 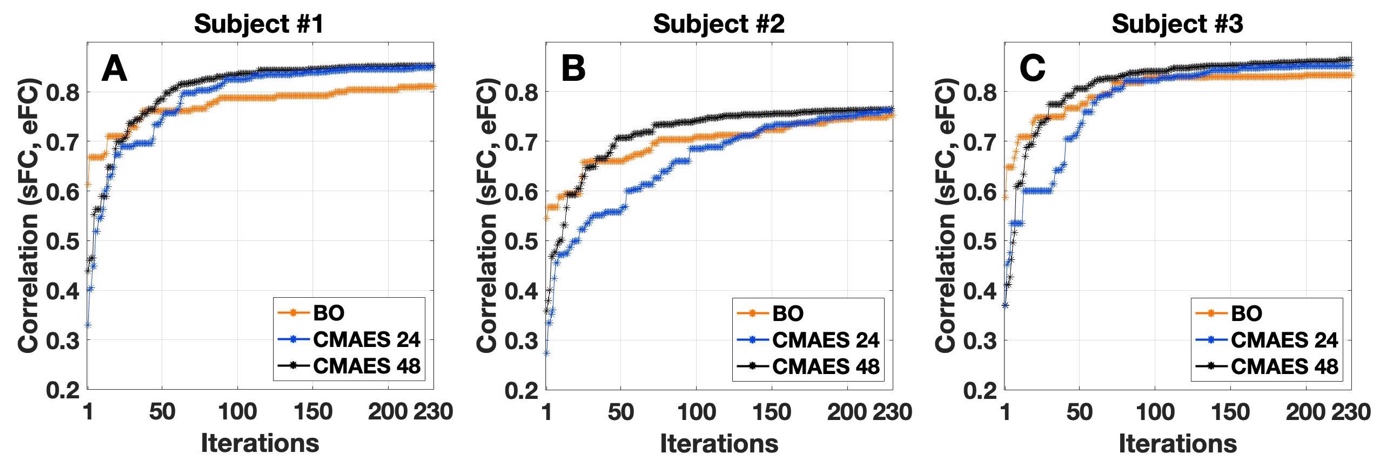 |
| --- |
| **Supplementary Fig 1.** **Examples of the algorithms’ convergence as given by the values of the goodness-of-fit versus the iteration number.** The plots show the gradual development of the obtained correlation between simulated and empirical FC (colored lines) for one run of the methods indicated in the legends (BO, CMAES 24 and CMAES 48, where the latter two correspond to an execution of the CMAES method with $\Lambda$ = 24 and $\Lambda$ = 48 candidate solutions in every iteration, respectively). The presented results pertain to the model parameter optimization in one of the considered high-dimensional parameter spaces (99 model parameters) for three randomly selected subjects, which are mentioned in the titles. This figure was created with MATLAB R2021a ([www.mathworks.com](http://www.mathworks.com)). |

-----------------------------------------------------------------------------------------------------------------

Here we provide some reasoning about the mathematical optimization algorithms (BO, CMAES) which we applied for model validation in the presented article.

*BO*

After an extensive testing of the C++ software package *BayesOpt* [[1](#_ENREF_1)], we worked with $\Lambda$ = 10 initial sampling points followed by 80 iteration steps in the low-dimensional cases (2D, 3D), see [[2](#_ENREF_2)] for additional illustrations and details about the tested criteria. The only difference compared to the setting from our previous work was that we selected the default value of $\Lambda$ = 10 sampling points also in 2D (instead of $\Lambda$ = 5). This did not cause considerable increases in computational costs, but made the simulations in the low-dimensional cases congruent prior to the intended comparisons with the high-dimensional ones (99D, 103D). In the latter, we selected $\Lambda$ = 200 initial samples with 230 subsequent iterations. Increasing $\Lambda$ became necessary for the establishment of a more plausible first approximation of the unknown goal function depending on such a high number of parameters. However, this served primarily as an initial orientation and, undoubtedly, the number of the following iteration steps had to be increased as well. Since a comparison with the results of a dense grid search was not possible, the choice of 230 steps was based on a tradeoff between computation time and the convergence of the observed fitting quality [**Supplementary Fig 1**]. We had to take into account that the supercomputer JURECA-DC [[3](#_ENREF_3)] enforces a simulation time limit of 24 hours for one job script. Similarly, as we could not compute a *success probability* [[2](#_ENREF_2)] to reach a fitting quality obtained via a grid search, we doubled the number of total algorithm executions for every subject from $R_{\text{max}}$ = 15 in our previous work to $R_{\text{max}}$ = 30 here. This appeared reasonable, because we could not anticipate the model behavior in high-dimensional parameter spaces beforehand. Further, the surrogate model for the goal function $F$ (i.e., correlation(sFC,eFC)) was updated after every single or after every 15^th^ new function evaluation in the low- and high-dimensional scenarios, respectively. This less frequent updating in the latter cases became necessary due to the increased time demands, which otherwise let our executed scripts (containing several algorithm executions running in parallel) come close to the simulation time limit of 24 hours.

*CMAES*

For the low-dimensional cases, we adapted the setting that we utilized and recommended previously [[2](#_ENREF_2)], i.e., 80 iterations with $\Lambda$ = 24 candidate solutions in every step. We highlight that this exceeded the recommended minimal sample size ($\Lambda$ = 6 in 2D and $\Lambda$ = 7 in 3D)*,* which typically scales with the number of parameters for optimization [[4](#_ENREF_4)]. Before applying the algorithm for model validation in the high-dimensional scenarios, we tested the values $\Lambda$ = 24 and $\Lambda$ = 48 for random test subjects. The latter case implies a doubled amount of invested computational resources compared to the former. (The recommended minimal sample size would be $\Lambda$ = 17 for both the 99D and the 103D case.) From our tests we concluded that, after approximately 150 iteration steps, a saturation of the observed fitting quality sets in frequently and the differences between the CMAES executions with $\Lambda$ = 24 and $\Lambda$ = 48 do not differ so much that the expenditures for the latter case would be justified [**Supplementary Fig 1**]. We therefore worked with $\Lambda$ = 24 candidate solutions in every iteration and also executed the CMAES method with this setting $R_{\text{max}}$ = 30 times to ensure comparability with BO.

-----------------------------------------------------------------------------------------------------------------

For a gradation of the intraclass correlation coefficient (ICC), we adapted the following table as suggested in [[5](#_ENREF_5), [6](#_ENREF_6)]:

| [0, 0.4[ | poor |
| --- | --- |
| [0.4, 0.6[ | fair |
| [0.6, 0.75[ | good |
| [0.75, 1[ | excellent |

-----------------------------------------------------------------------------------------------------------------

**Subject specificity of simulated connectivity and GoF (Methods)**

***Correlation-based variability and specificity of FC patterns***

To address the similarity of sFC matrices within and between subjects, we used the Pearson correlation of the vectorized matrices. More precisely, to compare two sFC matrices across different algorithm executions for the same subject and across different subjects, we randomly selected two subjects $s_{1},s_{2}$ ∈ {1, …, $S$}, $s_{1}\neq s_{2}$ and three execution indices

$q_{1},q_{2},q_{3}$ ∈ {1, …, $R_{\text{max}}$}, $q_{1}\neq q_{2}$. We then computed the correlation for the following sFC pairs:

- sFC ($s_{1},q_{1}$), sFC ($s_{1},q_{2}$): within subjects (intra-subject)
- sFC ($s_{1},q_{1}$), sFC ($s_{2},q_{3}$): between subjects (inter-subject)

This allowed us to investigate in how far the optimal sFC derived from several algorithm executions fluctuated within and across subjects. Overall, the discussed calculation of the similarity within and between subjects was repeated 1000 times in order to obtain adequate estimates of the underlying distributions.

For eFC, we also calculated the correlation-based similarity within and between subjects. A direct comparison with the sFC-related results could then be made for the correlations between subjects, i.e., eFC($s_{1}$), eFC($s_{2}$). For the variability within subjects, we utilized the data from the 4 available resting-state fMRI sessions per subject (provided by the HCP) to calculate different realizations of eFC as proxies for sFCs calculated from the repeated algorithm executions for the same subject. The respective indices $q_{1},q_{2},q_{3}$ were therefore randomly selected from these 4 fMRI sessions. This may however prevent a straightforward comparison of the fluctuations in eFC within subjects with those of sFC because the latter was always fitted to the same eFC calculated from the concatenated BOLD for the same subject.

Based on that, we also calculated the index of subject specificity of the simulated or empirical FC, which reflects the similarity of sFC or eFC within subjects relative to that between subjects [[7-9](#_ENREF_7)]. In this study, we considered 2 different ways of its calculation for FC:

1. Effect size (see later below) of statistical differences between the distributions of correlations within and between subjects;
2. Difference between the mean correlation within subjects and the mean correlation between subjects.

With this, we extended the specificity approach pursued in [[7](#_ENREF_7), [9](#_ENREF_9)], which was based on option 2 exclusively. Such a specificity measure is however based on the difference of the mean values only and does not take into account the other properties of the within- and between-subject distributions of similarity. We therefore put more focus on option 1.

***Difference-based variability and specificity of FC patterns and GoF***

Besides the Pearson correlation coefficient, which we used just above (and also as the sole measure for assessing the fitting quality of the model), we also quantified the similarity of two connectivity matrices $A,B$ ∈ $\mathbb{R}^{N\times N}$, $N$ ∈ $\mathbb{N}$ by the Frobenius norm $\left\| . \right\|_{\text{F}}$ of the difference matrix $C$ = $A-B$. The norm is defined as follows:

| $\left\Vert C \right\Vert_{\text{F}}=\sqrt{\sum_{i=1}^{N} \sum_{j=1}^{N} c_{ij}^{2}}=\sqrt{\sum_{i=1}^{N} \sum_{j=1}^{N} \left( a_{ij}-b_{ij} \right)^{2}}.$ | (4) |
| --- | --- |

We used both approaches (correlation and norm of the difference) as two stand-alone measures when investigating the FC variability within and between subjects, i.e., across different algorithm executions and fMRI measurements for sFC and eFC, respectively, for the same subject and across different subjects, accordingly. Note that this pertains only to a comparison of sFC with sFC or eFC with eFC, but not across modalities.

We stress here that in the norm-based approach, we applied the Fisher $z$-transformation to the elements of the sFCs (and eFCs) to render them normally distributed before computing the differences. The Fisher $z$-transformation is defined as follows:

| $z = \text{artanh}(r) = \frac{1}{2}\ln\left( \frac{1+r}{1-r} \right) \text{for} \left\vert r \right\vert<1.$ | (5) |
| --- | --- |

It is typically used when statistical tests are intended to be applied on arithmetic operations of correlations, which are not interval-scaled and often show a skewed distribution when close to 1 or -1 [[10](#_ENREF_10)].

The same procedure of a random selection of subjects and indices of optimization runs/fMRI measurements, which was employed for the correlation-based similarity above, was also used for the similarity based on the matrix differences. Accordingly, we calculated the index of subject specificity of the simulated or empirical data based on the FC differences:

1. Effect size of statistical differences between the distributions of the $\left\| . \right\|_{\text{F}}$-norms of the (Fisher $z$-transformed FC matrix) differences between and within subjects;
2. Difference between the mean $\left\| . \right\|_{\text{F}}$-norm of the differences between subjects and the mean $\left\| . \right\|_{\text{F}}$-norm of the differences within subjects.

Here, we also concentrated more on option 1 featuring the effect sizes. Since the norm of the calculated (within- or between-subject) matrix differences is a measure of sFC (eFC) dissimilarity, the positions of the intra- and inter-subject difference distributions were exchanged as compared with those for the FC correlations. This guarantees a positive specificity index in the case when the intra-subject variability is smaller than inter-subject one, as expected.

Additionally, we calculated the subject specificity for the GoF values:

1. Effect size of statistical differences between (Fisher $z$-transformed) absolute GoF differences between and within subjects.

In summary, we computed the FC variations, i.e., correlations and difference norms, both within and between subjects for the repeated parameter optimizations. This allowed us to calculate several measures of reliability and subject specificity pertaining to the observed differences (within subjects vs. between subjects). We wanted to assess the variability of sFC patterns across subjects for all tested dimensions of the parameter space. The same applies to the fitting quality (GoF), for which we also calculated a related measure. We were eager to investigate the impact of a varying number of free model parameters on the simulation outcomes.

-----------------------------------------------------------------------------------------------------------------

For the effect size (ES) of statistical differences computed with Rosenthal’s formula [[11](#_ENREF_11)], we can adapt the gradation suggested in [[12](#_ENREF_12)]:

| [0, 0.2[ | very low |
| --- | --- |
| [0.2, 0.4[ | low |
| [0.4, 0.6[ | moderate |
| [0.6, 0.8[ | strong |
| [0.8, 1] | very strong |

-----------------------------------------------------------------------------------------------------------------

| \|  \| **Sch10000** \| \| \| **HO0Thr** \| \| \| \| --- \| --- \| --- \| --- \| --- \| --- \| --- \| \| *2D – 3D* \| *3D – 103D* \| *2D – 103D* \| *2D – 3D* \| *3D – 99D* \| *2D – 99D* \| \| **BO** \| 0.30 \| 0.78 \| 0.79 \| 0.32 \| 0.80 \| 0.82 \| \| **CMAES** \| 0.41 \| 0.59 \| 0.75 \| 0.39 \| 0.64 \| 0.77 \| |
| --- | --- | --- | --- | --- | --- | --- | --- | --- | --- | --- | --- | --- | --- | --- | --- | --- | --- | --- | --- | --- | --- | --- | --- | --- | --- | --- | --- |
| **Supplementary Table 2.** **Intensity of the goodness-of-fit (GoF) changes across parameter spaces.** For the GoF distributions shown in **Fig 1** of the main text, the values in the table indicate the effect size (ES) of the GoF increases observed for the transitions from one to another parameter space. All numbers were calculated with Rosenthal’s formula [[11](#_ENREF_11)]. The notations in the second line of the table, for example, “*2D – 3D”* indicate the two parameter space dimensions used for comparison. Results are shown for both considered optimization algorithms (BO, CMAES) and atlases (Sch100, HO0Thr). |

-----------------------------------------------------------------------------------------------------------------

| 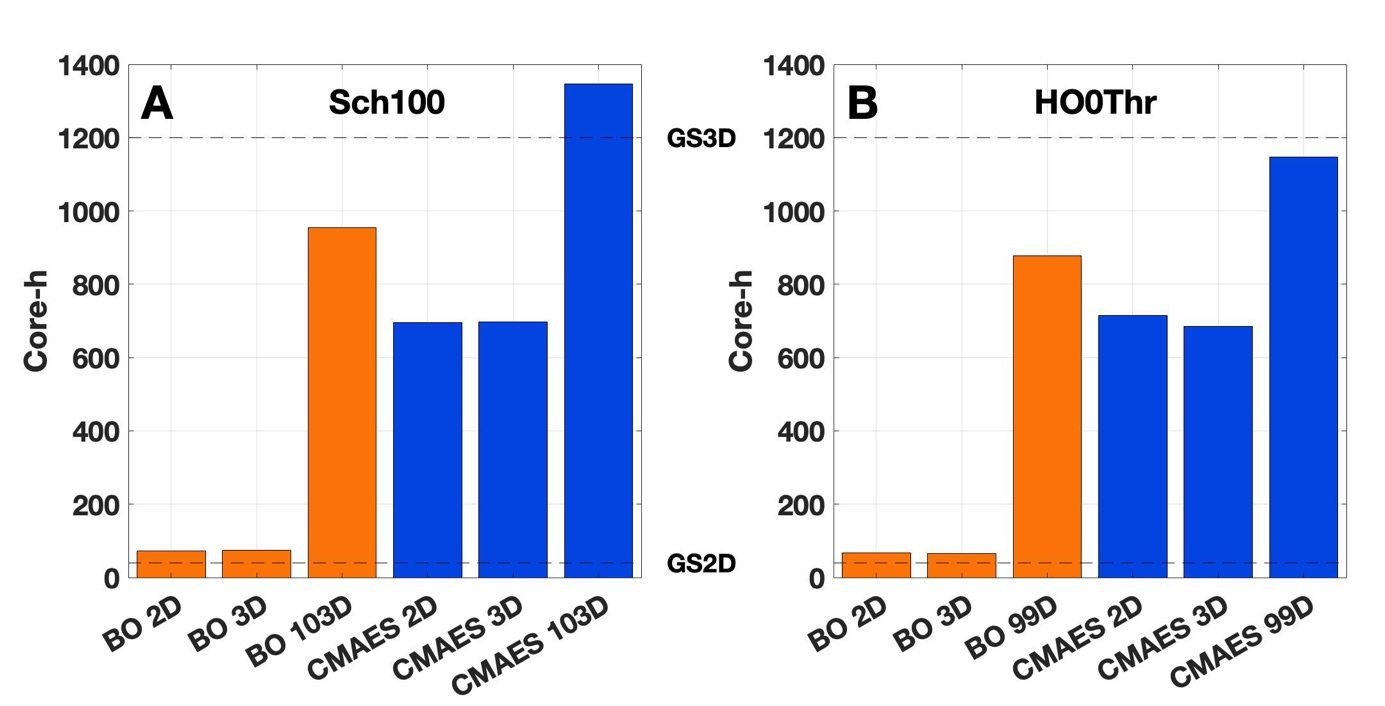 |
| --- |
| **Supplementary Fig 3.** **Median resource consumption for the execution of the optimization algorithms.** The considered methods and parameter spaces (BO 2D, CMAES 2D, BO 3D and CMAES 3D in the low-dimensional cases and BO 103D, CMAES 103D, BO 99D and CMAES 99D in the high-dimensional ones) are provided on the horizontal axes for **(A)** the Schaefer atlas (Sch100) and **(B)** the Harvard-Oxford atlas (HO0Thr). On the vertical axes, the amounts of invested core-hours are given. Bars show the median computational requirements for 30 algorithm executions per subject. Dashed horizontal lines indicate the approximate resource consumption of a comparable grid search in the two- and three-dimensional parameter spaces (indicated by GS2D and GS3D, respectively) [[2](#_ENREF_2)]. This figure was created with MATLAB R2021a ([www.mathworks.com](http://www.mathworks.com)). |

-----------------------------------------------------------------------------------------------------------------

| 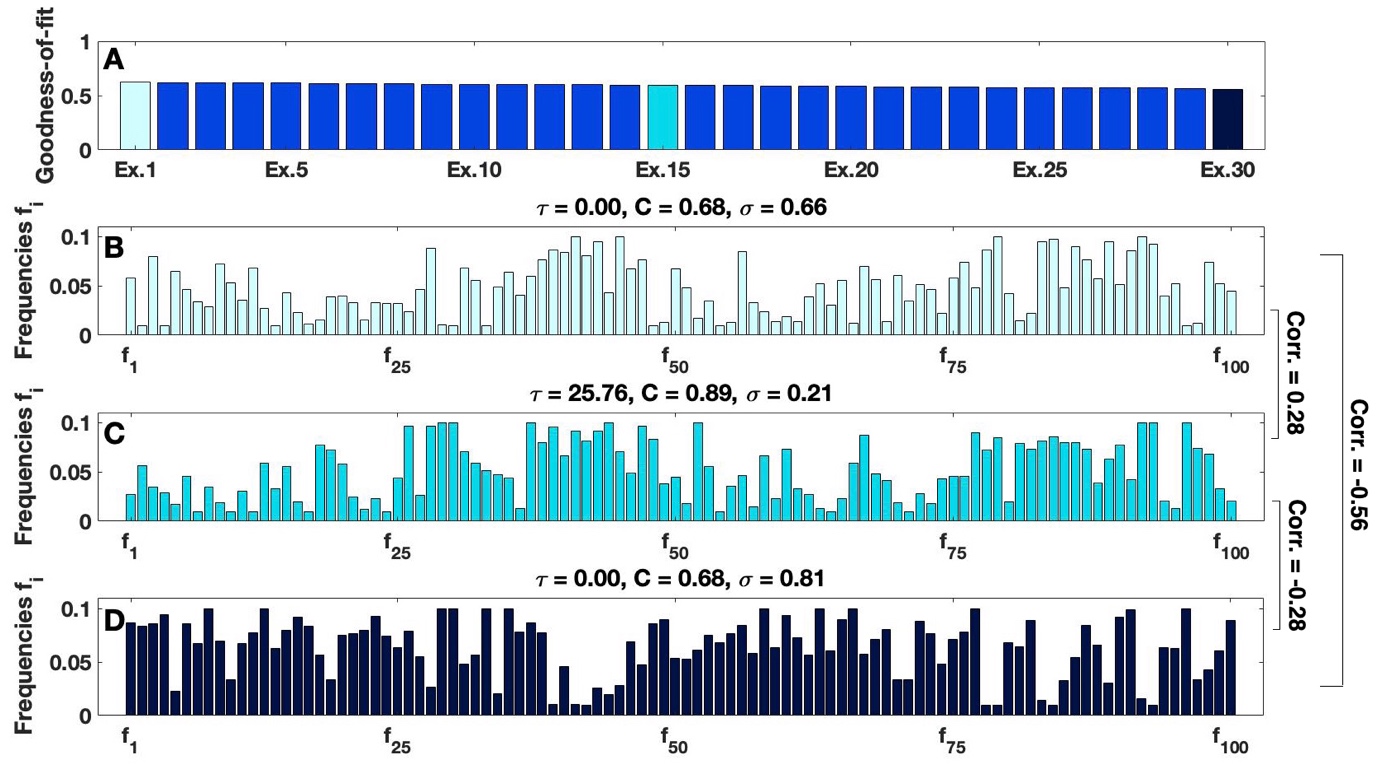 |
| --- |
| **Supplementary Fig 4.** **Example of the goodness-of-fit (GoF) and optimized model parameter values in the 103-dimensional parameter space (same conditions as in Fig 2 of the main text).** **(A)** Bars indicate the GoF values detected in 30 executions (Ex.) of the CMAES algorithm for one subject. The executions were sorted in descending order, based on the observed model fitting values. Light blue, cyan and dark blue bars highlight the highest, middle and lowest fitting quality, respectively. **(B-D)** The values for delay $\tau$, coupling $C$ and noise intensity $\sigma$ provided in the titles together with color-matched bar plots showing the values of the model parameters which led to the selected GoF values highlighted in **(A)**, i.e., using the parameters illustrated in **(B)**, **(C)** and **(D)** as input for the model simulations resulted in the highest, middle and lowest observed fitting quality, respectively. The correlations (Corr.) between the $N$-dimensional vectors $\left( f_{i} \right)_{1\leq i\leq N}$ of optimized frequencies for the presented executions are written vertically next to the plots **(B)**, **(C)** and **(D)**. This figure was created with MATLAB R2021a ([www.mathworks.com](http://www.mathworks.com)). |

-----------------------------------------------------------------------------------------------------------------

We evaluated the variability (interquartile range, IQR) of the approximated optimal model parameters and GoF values across algorithm executions for all subjects. Lower values of the IQR reflect smaller intra-subject variability. For the sake of visual comparability of the spreads of parameters, which varied along values of different orders of magnitude (e.g., $\tau$ ∈ [0, 100] and $\sigma$ ∈ [0, 2]), we divided the computed IQR values by the maximal value that the respective parameter could reach as specified by the considered parameter ranges (1 for GoF). This approach delivered the illustration closest to the unscaled data treated in separate parameter ranges. We stress that a division by the median might distort the illustration in cases where the median of optimal values is (close to) zero. When optimized with the CMAES method for the Schaefer atlas, the parameters of delay $\tau$ show a relatively broad spread of fluctuations across optimization trials for all subjects. The scaled (by maximum parameter value) IQR is distributed around its median of approximately 0.20 [**Supplementary Fig 5A**]. However, the boxplot including whiskers covers the entire range from 0 to 1, meaning that very high as well as very low fluctuations of delay values may frequently be observed for a certain portion of subjects. For the Harvard-Oxford atlas, the spread of IQR values appears to be somewhat lower together with zero median. Unlike CMAES, the BO method shows a similar spread of the optimized delay $\tau$ fluctuations for both considered atlases, where the distributions of the scaled IQR are centered around medians in the range of 0.43-0.46 [**Supplementary Fig 5**]. This indicates that the extent of fluctuations within subjects tends to be on a nearly constant level for BO. A persistent variability can be observed for the frequency parameters$\left( f_{i} \right)_{1\leq i\leq N}$ optimized by CMAES, where (i) the scaled median IQR remains between 0.22 and 0.91 across brain regions, and (ii) the spreads of the frequencies obtained for individual subjects concentrate narrowly around their medians in most cases [**Supplementary Fig 5**, blue boxes for the frequencies $\left( f_{i} \right)_{1\leq i\leq N}$]. The situation is much more heterogeneous for the BO method, especially for the Harvard-Oxford atlas [**Supplementary Fig 5B**, orange boxes for the frequencies $\left( f_{i} \right)_{1\leq i\leq N}$], where the scaled median IQR of the optimized frequency parameters can be very different across brain regions. Furthermore, the individual subjects may also exhibit a rather distinct frequency spread and broadly deviate from their scaled median IQR for many brain regions. The other model parameters of coupling $C$ and noise intensity $\sigma$ demonstrate much more consistent optimization results with narrow distributions of the scaled IQR across trials and subjects with medians between 0.06 and 0.15 [**Supplementary Fig 5**]. Most reproducible are however the GoF values, where the median of the scaled IQR is close to 0.05 (Sch100) and 0.02 (HO0Thr) for both considered optimization methods [**Supplementary Fig 5**], despite the broadly distributed (delay and) frequency parameters.

| 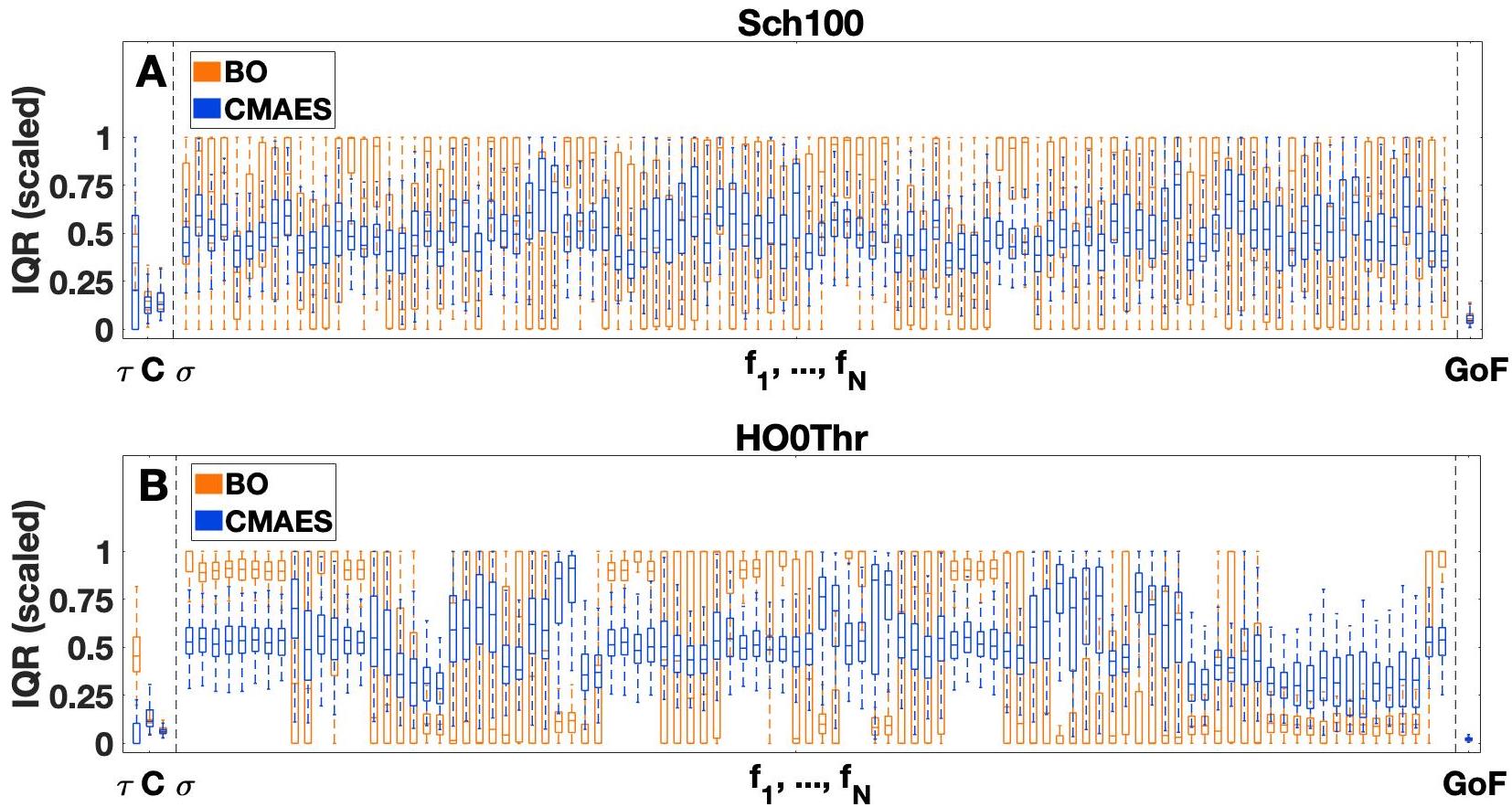 |
| --- |
| **Supplementary Fig 5.** **Variability of the optimized model parameters and GoF values across algorithm executions for all subjects.** The considered model parameters (delay $\tau$, coupling $C$, noise intensity $\sigma$, frequencies $\left( f_{i} \right)_{1\leq i\leq N}$ and also GoF) are indicated on the horizontal axes along with the values of their scaled interquartile range (IQR) on the vertical axes. For every subject, the IQRs of the model parameters and GoF values found in 30 optimization executions with random initial data were computed and then separately divided by the respective, highest possible parameter values as specified by the considered parameter ranges. Boxplots visualize the resulting, visually comparable distributions of the scaled IQRs of all parameters (including GoF) for the optimization approaches based on BO (orange boxes) and CMAES (blue boxes). The results pertain to **(A)** the Schaefer atlas (Sch100) and **(B)** the Harvard-Oxford atlas (HO0Thr). This figure was created with MATLAB R2021a ([www.mathworks.com](http://www.mathworks.com)). |

-----------------------------------------------------------------------------------------------------------------

| 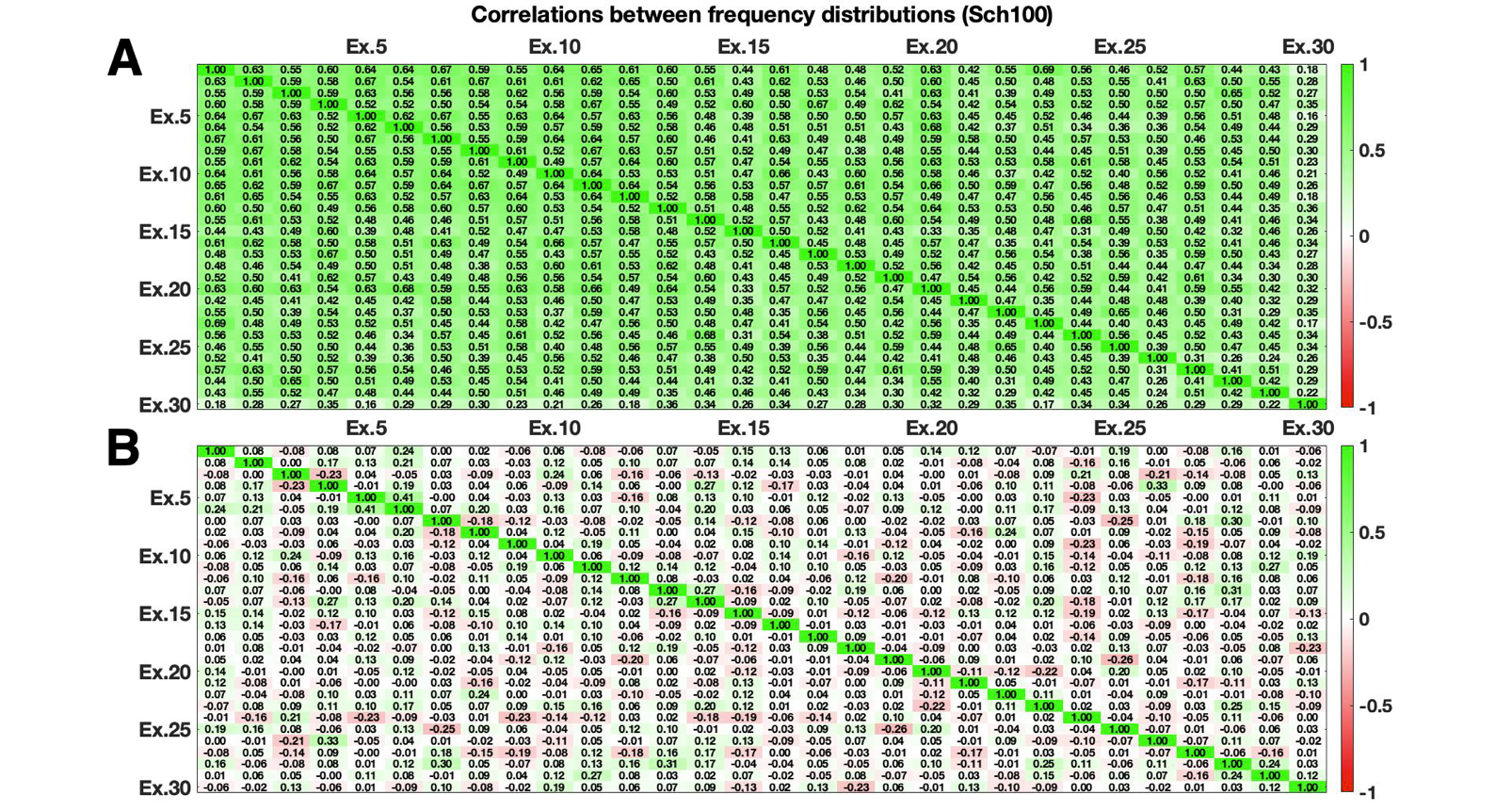 |
| --- |
| **Supplementary Fig 6.** **Examples of the correlations between optimized frequency parameter sets (cf. Fig 3A of the main text) for two selected subjects (shown in (A) and (B), respectively).** For every subject, the correlations between any two sets of optimized frequency parameters $\left( f_{i} \right)_{1\leq i\leq N}$ obtained in two algorithm executions (Ex.) with random initial data were correlated and then indicated in the cells which are highlighted in color. The numbers presented here pertain to the model validation performed in the 103-dimensional parameter space via BO for Schaefer’s atlas (Sch100), see also **Fig 3F** of the main text. This figure was created with MATLAB R2021a ([www.mathworks.com](http://www.mathworks.com)). |

-----------------------------------------------------------------------------------------------------------------

| **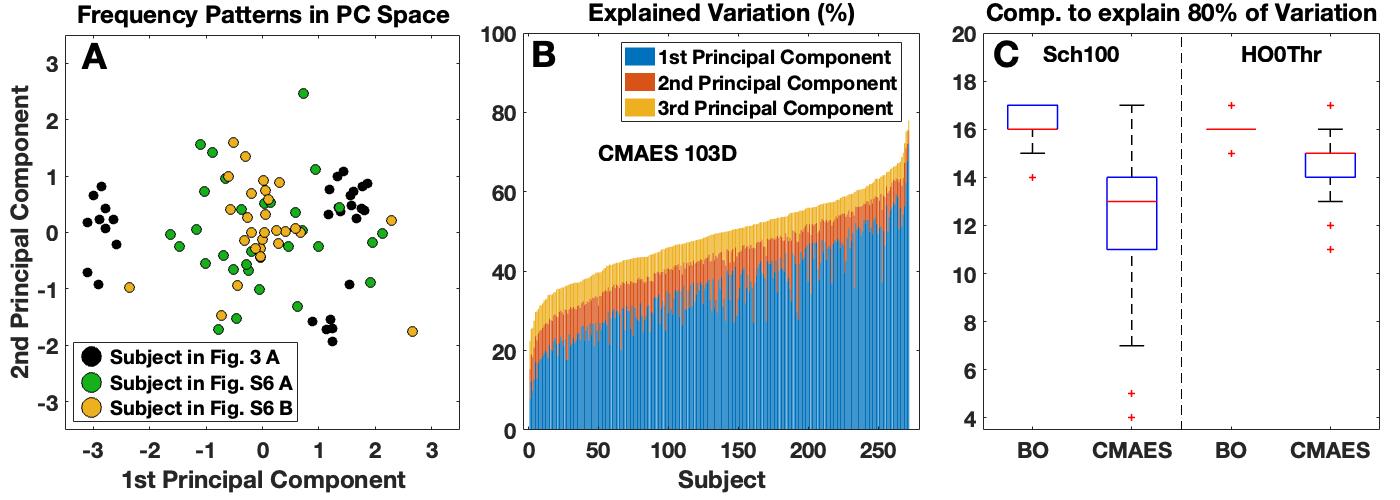** |
| --- |
| **Supplementary Fig 7. Principal component analysis (PCA) results for the optimized model parameters. (A)** Scatter plots visualize the PC scores of the optimized frequency parameters $\left( f_{i} \right)_{1\leq i\leq N}$derived from 30 algorithm executions. Results are shown in the PC space spanned by the first two principal components indicated on the axes. Black, green and sand-colored dots represent the values for three subjects. The same subjects were also used as examples in **Fig 3** of the main text as well as in **Supplementary Fig 6**. **(B)** Color-stacked bar plots show the fraction of variation explained by the first three principal components for all subjects. The numbers of the subjects are provided on the horizontal axis along with the percentages of explained variation on the vertical axis. As indicated in the plot, the parameter optimization was performed via CMAES in the 103-dimensional parameter space (Sch100). **(C)** Boxplots visualize the distributions of principal components (Comp.) required to explain at least 80% of variation in the optimized frequency parameters for all considered high-dimensional cases. The names of the utilized algorithms are provided on the horizontal axis along with the number of components on the vertical axis. A dashed line separates the results for the different atlases (Sch100, HO0Thr) indicated in the plot. This figure was created with MATLAB R2021a ([www.mathworks.com](http://www.mathworks.com)). |

-----------------------------------------------------------------------------------------------------------------

| **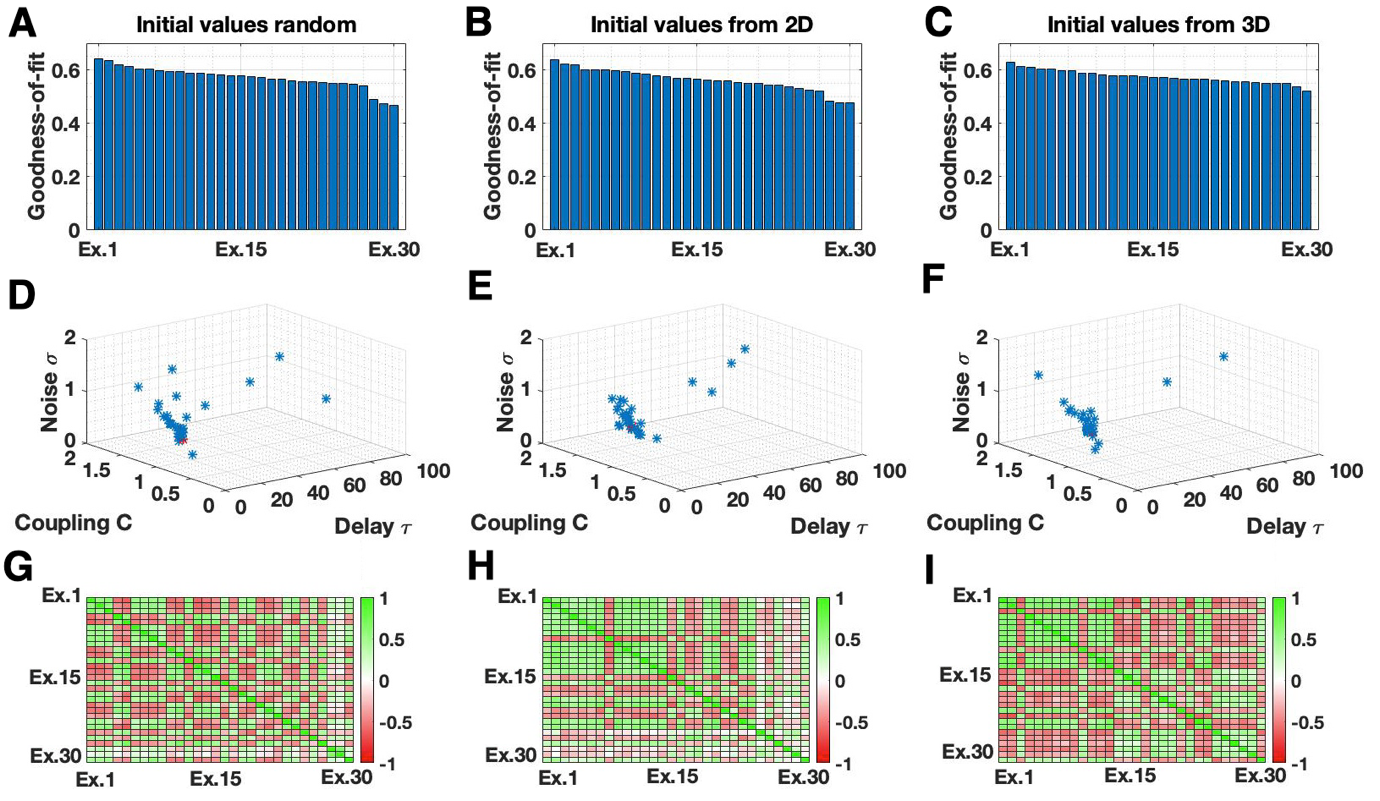** |
| --- |
| **Supplementary Fig 8. Examples of the model fitting results for different starting points in the 103-dimensional parameter space.** For one subject, the CMAES method was executed 30 times, where the parameter values for the initial search distribution mean were taken **(A,D,G)** randomly and uniformly from the respective parameter intervals (see Methods); **(B,E,H)** from the results of parameter optimization in the 2D space; and **(C,F,I)** from the results of parameter optimization in the 3D space. For the latter two cases, the parameters of noise intensity and frequencies in 2D as well as frequencies in 3D had the initial values that were considered in the corresponding low-dimensional model validations (e.g., $\sigma$ = 0.3 in 2D, see Methods). **(A-C)** Bars indicate the goodness-of-fit (GoF) values detected in 30 algorithm executions (Ex.). The executions were sorted in descending order, based on the observed model fitting values. **(D-F)** Blue asterisks show the location of the approximated optimal parameters of coupling, delay and noise intensity in the 3D subspace of the 103D parameter space. The configuration yielding the highest GoF is marked in red (may be covered by other solutions). **(G-I)** Correlations between optimized frequency parameter sets for different optimization runs (Ex.) (cf. **Fig 3A** of the main text, **Supplementary Fig 6**). This figure was created with MATLAB R2021a ([www.mathworks.com](http://www.mathworks.com)). |

-----------------------------------------------------------------------------------------------------------------

| 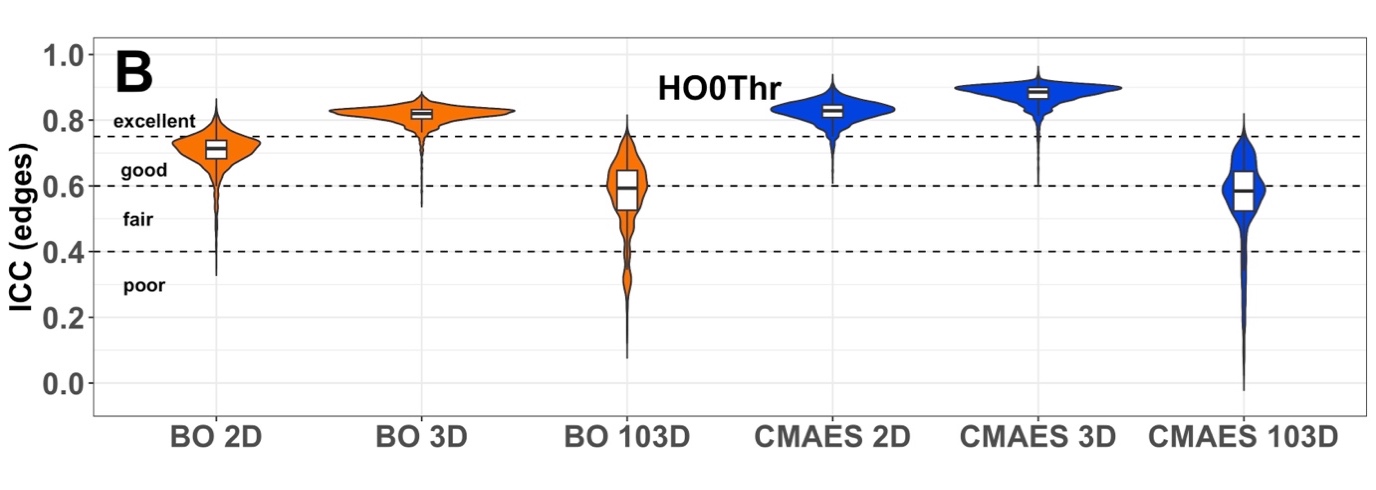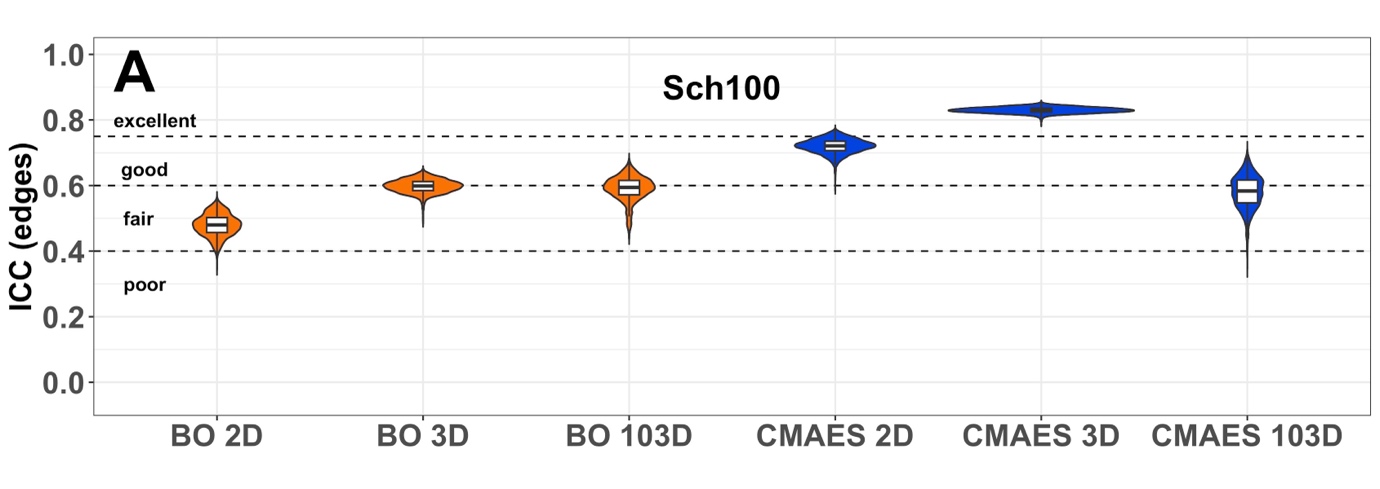 |
| --- |
| **Supplementary Fig 9. Reliability of the matrix edges for all considered conditions.** Violins show the distributions of the intraclass correlation coefficients (ICC) for the off-diagonal edges of the matrices of sFC in the low- and high-dimensional cases. Dashed horizontal lines indicate the levels of *poor*, *fair*, *good* and *excellent* reliability in terms of the ICC as suggested in [[5](#_ENREF_5), [6](#_ENREF_6)]. The presented distributions reflect the results obtained during the model validation for **(A)** the Schaefer atlas (Sch100) and **(B)** the Harvard-Oxford parcellation (HO0Thr). The employed optimization approaches are listed on the horizontal axes along with the ICC scores of the sFC edges on the vertical axes. |

-----------------------------------------------------------------------------------------------------------------

**Subject specificity of simulated connectivity and GoF (Results)**

This section is dedicated to a deeper investigation of those two modeling results that turned out to be the most reliable ones across optimization runs, namely the sFC and GoF.

***Correlations between connectivity patterns***

We compared the sFC matrices obtained for different executions of the optimization methods. The corresponding matrix correlations were calculated within (intra) and between (inter) subjects (see Methods above). Here we found that the sFC correlations within subjects are higher (sFC is more similar) than those between subjects, which is consistent across the considered optimization methods and brain atlases [**Supplementary Fig 10**]. For the Schaefer atlas, however, the inter-subject sFC correlations display relatively broad distributions (IQR on average around 228% higher) as compared with those for the Harvard-Oxford atlas [**Supplementary Fig 10**]. This may indicate a non-uniform inter-individual variability of sFC for the former atlas, where the sFC correlations between subjects are widely spread from small to large values.

The discussed correlations can also be calculated for the eFC. A few eFC realizations for the same subject are extractable from the repeated fMRI measurements in the considered HCP dataset (see Methods above). The distributions of the intra- and inter-subject eFC correlations are illustrated in **Supplementary Fig 10** (black boxes in the leftmost sections) indicating a higher intra-subject eFC similarity as compared to that between subjects.

| 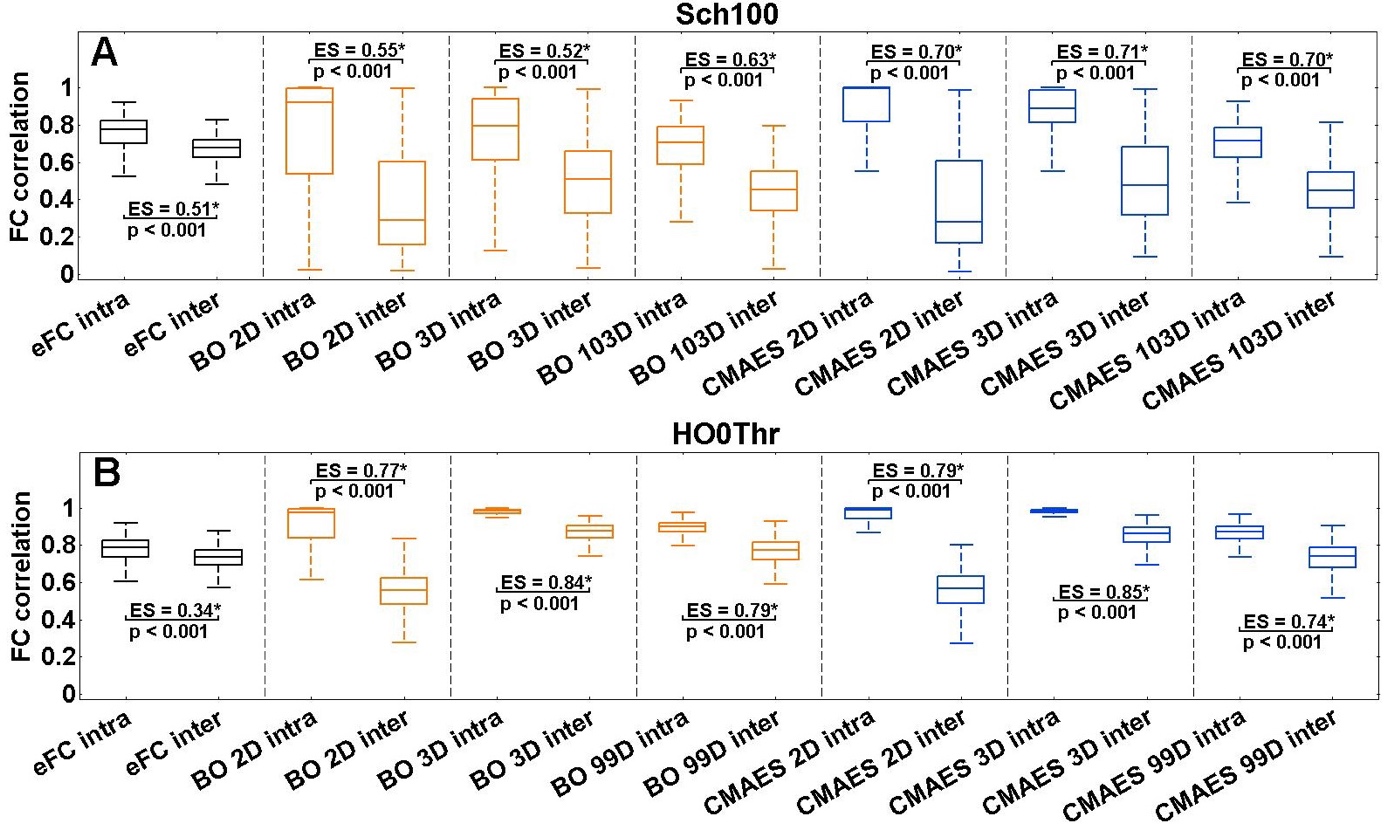 |
| --- |
| **Supplementary Fig 10.** **Correlations of the FC matrices within and between subjects.** For two randomly selected different subjects, two different algorithm solutions for one subject together with one solution for the other subject were randomly chosen in order to compute the correlations of any two sFC matrices, i.e., within (intra) and between (inter) subjects, respectively (see Methods above). For the correlations of eFC, data from the four available resting-state fMRI sessions per subject were considered. This procedure was repeated 1000 times. Boxplots show the distributions of the resulting matrix correlations for the algorithms and parameter spaces listed on the horizontal axes. The correlations presented on the vertical axes pertain to **(A)** the Schaefer atlas (Sch100) and **(B)** the Harvard-Oxford atlas (HO0Thr). Outliers are not shown in this figure. The effect sizes (ES) of the deviations between the intra- and inter-subject distributions of the FC correlations in all considered parameter spaces are indicated in the plots together with $p$-values of the Wilcoxon rank-sum test. Statistically significant differences are marked with an asterisk. The significance level of 5%, $p$ < 0.05, has been Bonferroni-corrected for multiple comparisons. This figure was created with MATLAB R2021a ([www.mathworks.com](http://www.mathworks.com)). |

-----------------------------------------------------------------------------------------------------------------

An important property that can be calculated from the intra- and inter-subject FC (dis)similarities is the *subject specificity.* It reflects whether the results are more similar to each other within than between subjects. We characterize the subject specificity of FCs principally by the ES between the distributions of their intra- and inter-subject correlations (see Methods above). The specificity indices derived from the intra- and inter-subject correlations of sFCs are highest in the high-dimensional case for the Schaefer atlas with BO, but remain almost constant across parameter spaces for CMAES [**Supplementary Fig 11**]. For the Harvard-Oxford atlas, both algorithms seem to be characterized by a specificity from the same narrow range of values for all dimensions [**Supplementary Fig 11**]. Further, the model simulations generated connectivity matrices which are more subject-specific than the empirical ones for both atlases and optimization methods [**Supplementary Fig 10**].

We however note here that the calculated intra- and inter-subject variability and subject specificity of eFC and sFC cannot always be directly compared to each other. Indeed, for sFC, these quantities were calculated for different runs of the optimization methods, where the sFC was always fitted to the same eFC of a given subject. This may allow for a comparison of the inter-subject similarity and variability of eFC and sFC. It however prevents a straightforward comparison of the intra-subject variability and thus subject specificity of eFC and sFC, unless we assume that the repeated model simulations, fittings and sFC optimizations for different initial conditions may correspond to separate (fMRI) measurements of empirical data. We also note here that the observed low inter-subject correlations of sFC relative to those for eFC [**Supplementary Fig 10**] may indicate that the considered modeling approach enhances the inter-individual variability of the functional connectomes.

An additional measure of the subject specificity based on the differences of the mean values (see Methods above) is illustrated in **Supplementary Fig 11**. Only for a few considered cases, we may observe some monotonic decline of the subject specificity for sFC with increasing dimension of the parameter space, such that the illustrated results to a large extent agree with those discussed above. We however note here that such a specificity measure based on the mean values only does not take into account the other properties of the intra- and inter-subject similarity distributions, in contrast to the ES-based specificity index.

| **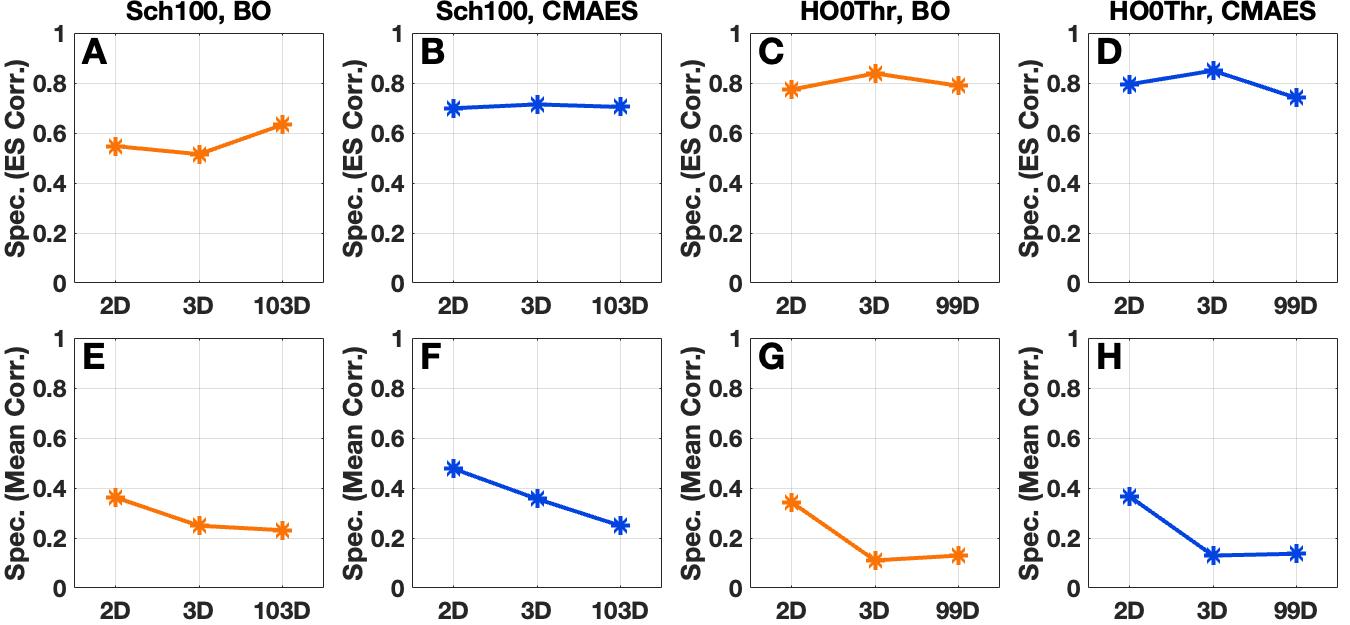** |
| --- |
| **Supplementary Fig 11.** **Subject specificity derived from the differences between the intra- and inter-subject distributions of similarities of sFCs obtained after several executions of the optimization algorithms.** The sampling approach was chosen as in **Supplementary Fig 10**. The considered atlases and methods are provided in the titles of the plots, while the parameter spaces are listed on the horizontal axes. **(A-D)** Specificity indices defined as the effect sizes (ES) between the distributions of the intra- and inter-subject correlations of sFCs (Spec. (ES Corr.)). **(E-H)** Specificity indices based on the differences between the mean values of the intra- and inter-subject distributions of sFC correlations (Spec. (Mean Corr.)). This figure was created with MATLAB R2021a ([www.mathworks.com](http://www.mathworks.com)). |

-----------------------------------------------------------------------------------------------------------------

***Differences between connectivity patterns***

The situation is very similar when the resemblance of the FC matrices within and between subjects is evaluated by the matrix differences measured by the Frobenius norm $\left\| . \right\|_{\text{F}}$ (see Methods above). Analogously, the intra-subject sFC differences appear to be lower than the inter-subject differences [**Supplementary Fig 12**]. We also observed the patterns of broad distributions of the sFC similarity for the Schaefer atlas, where the IQR of the inter-subject sFC differences is on average around 108% larger than that for the Harvard-Oxford atlas [**Supplementary Fig 12**].

We found a relatively high subject specificity of sFC for all considered conditions, where the ES ranges from moderate to strong with somewhat stronger results for the Harvard-Oxford atlas [**Supplementary Fig 12**]. For the Schaefer atlas, we observed a drop in the specificity accompanying the transition to the high-dimensional cases for CMAES [**Supplementary** **Figs 12,13**]. For the other conditions, no such clear trend can be reported, because the specificity values are gathered in a narrow range for all dimensions of the parameter space [**Supplementary** **Figs 12,13**]. Therefore, increasing the dimension of the model parameter space may not consistently lead to a reduction or an enhancement of the subject specificity of sFC.

For the specificity based on the differences of mean values, we observed only very small changes across parameter spaces [**Supplementary Fig 13**]. We stress that the ES-based approach might be a more appropriate specificity measure especially in the cases of heterogeneous intra- and inter-subject variability of similarity values as in our study [**Supplementary Figs 10,12**].

The reported findings thus demonstrate that only for the Schaefer atlas, CMAES and difference-based similarity of sFC, we may observe a considerable drop in the subject specificity for model fitting in a high-dimensional parameter space [**Supplementary Fig 13**]. For the other considered conditions, the extent of the specificity varied only slightly across parameter dimensions with a possible small enhancement for more free parameters in some cases.

We also calculated the specificity of the GoF values (see Methods above). For this quantity, we observed a monotonically decreasing trend for the Schaefer atlas as the dimension of the model parameter space increases. The specificity for the Harvard-Oxford atlas, however, does not consistently decay for more free parameters, such that the high-dimensional model validation does not necessarily imply a lower subject specificity here either [**Supplementary Fig 13**].

| 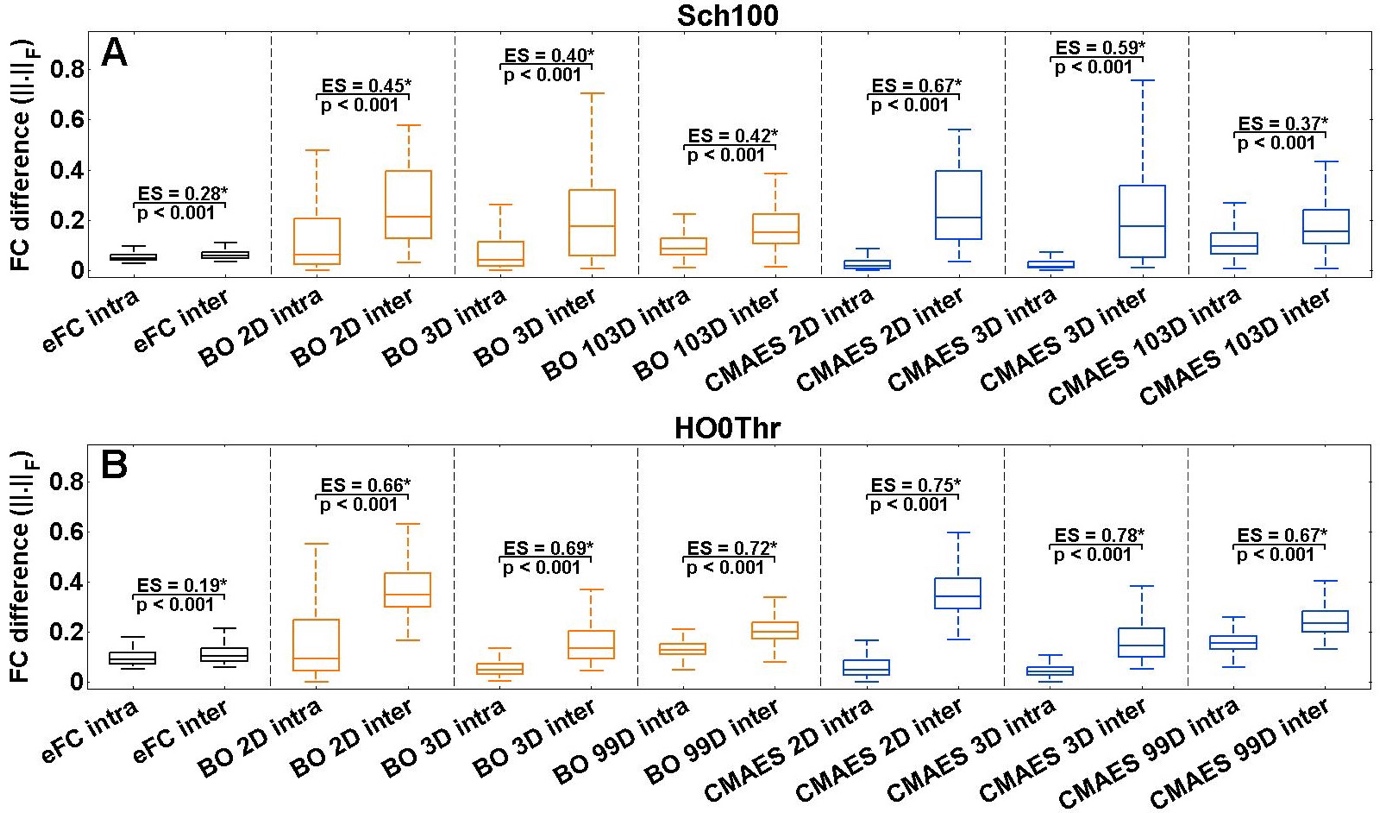 |
| --- |
| **Supplementary Fig 12.** **Differences of the FC matrices within and between subjects.** The same approach and notations as in **Supplementary Fig 10** were used, but for the Frobenius norm $\left\Vert. \right\Vert_{\text{F}}$ of the differences of any two (Fisher-transformed) FC matrices. The differences presented on the vertical axes were normalized by one maximum value calculated separately for each atlas. The ES was calculated between inter- and intra-subject distributions of FC correlations, see Methods above. This figure was created with MATLAB R2021a ([www.mathworks.com](http://www.mathworks.com)). |

-----------------------------------------------------------------------------------------------------------------

| **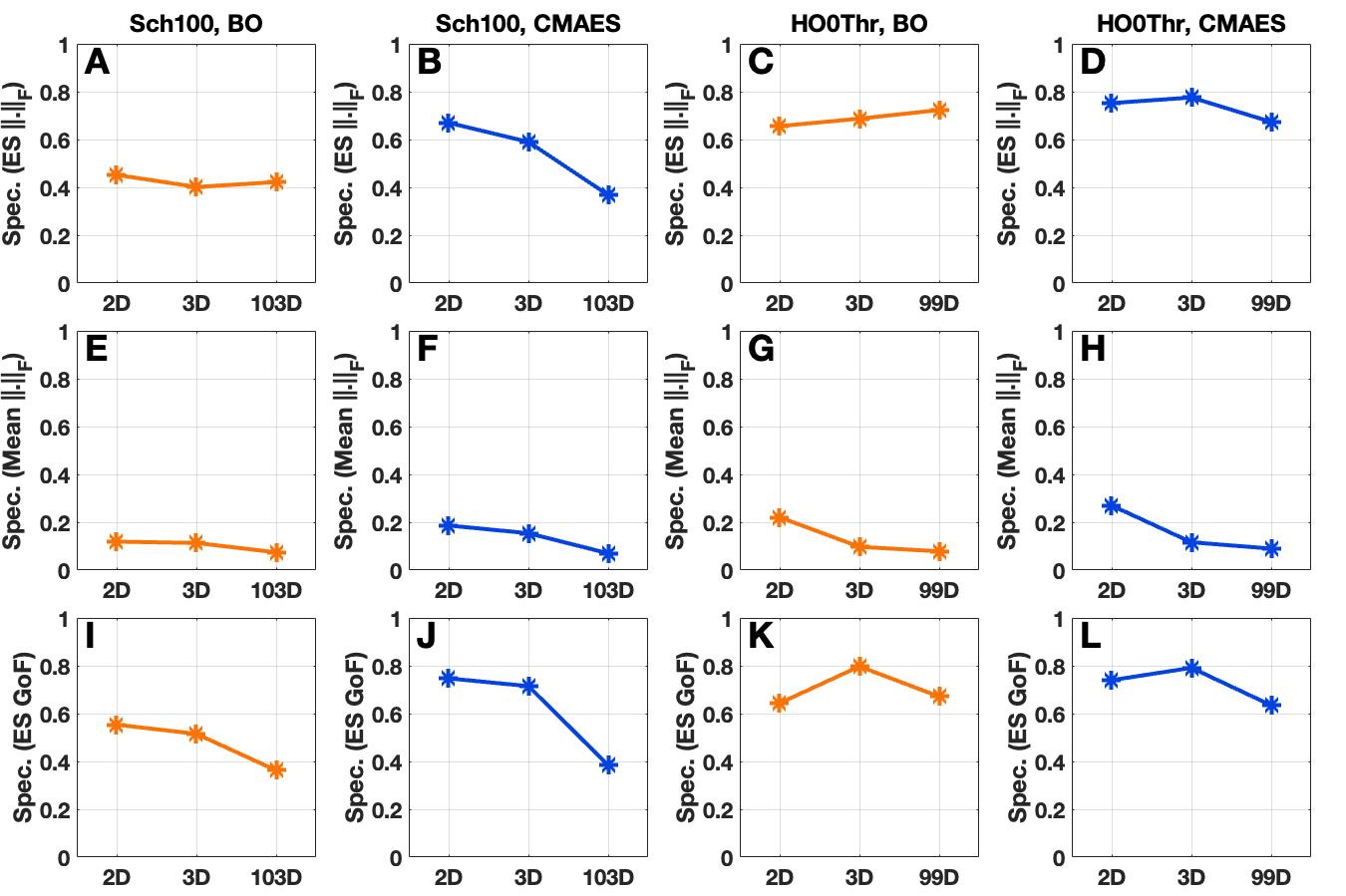** |
| --- |
| **Supplementary Fig 13.** **Subject specificity based on the differences between the inter- and intra-subject similarity distributions of sFC and GoF values obtained after several executions of the optimization algorithms.** The sampling approach was chosen as in **Supplementary** **Fig 10**. The considered atlases and methods are provided in the titles of the plots, while the parameter spaces are listed on the horizontal axes. **(A-D)** Specificity indices defined as the ES between the distributions of the inter- and intra-subject sFC differences measured by the Frobenius norm (Spec. (ES $\left\Vert. \right\Vert_{\text{F}}$)). Since the calculated matrix difference is a measure of sFC dissimilarity, the positions of the intra- and inter-subjects difference distributions were exchanged in order to obtain positive ES, see Methods above. **(E-H)** Specificity indices derived from the differences between the mean values of inter- and intra-subject sFC differences measured by the Frobenius norm (Spec. (Mean $\left\Vert. \right\Vert_{\text{F}}$)). **(I-L)** GoF-based specificity defined as the ES between the inter- and intra-subject differences of GoF values. This figure was created with MATLAB R2021a ([www.mathworks.com](http://www.mathworks.com)). |

-----------------------------------------------------------------------------------------------------------------

| 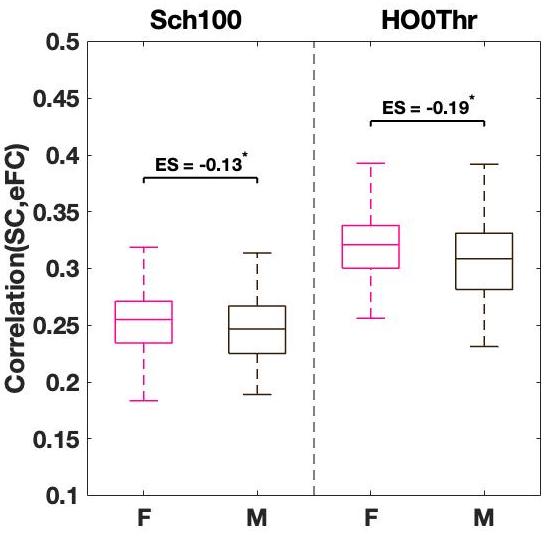​​​​​​​​​​​​​​ |
| --- |
| **Supplementary Fig 14. Sex differences in empirical data.** Colored boxplots visualize the distributions of the correlations between SC and eFC (structure-function relationship) separately for males (M, brown) and females (F, pink). A solid vertical line segregates the outcomes for the Schaefer atlas (Sch100) and the Harvard-Oxford parcellation (HO0Thr). The effect sizes (ES) of the sex differences are indicated in the plot. Results that proved statistically significant in the Wilcoxon rank-sum test are marked with an asterisk. This figure was created with MATLAB R2021a ([www.mathworks.com](http://www.mathworks.com)). |

-----------------------------------------------------------------------------------------------------------------

| 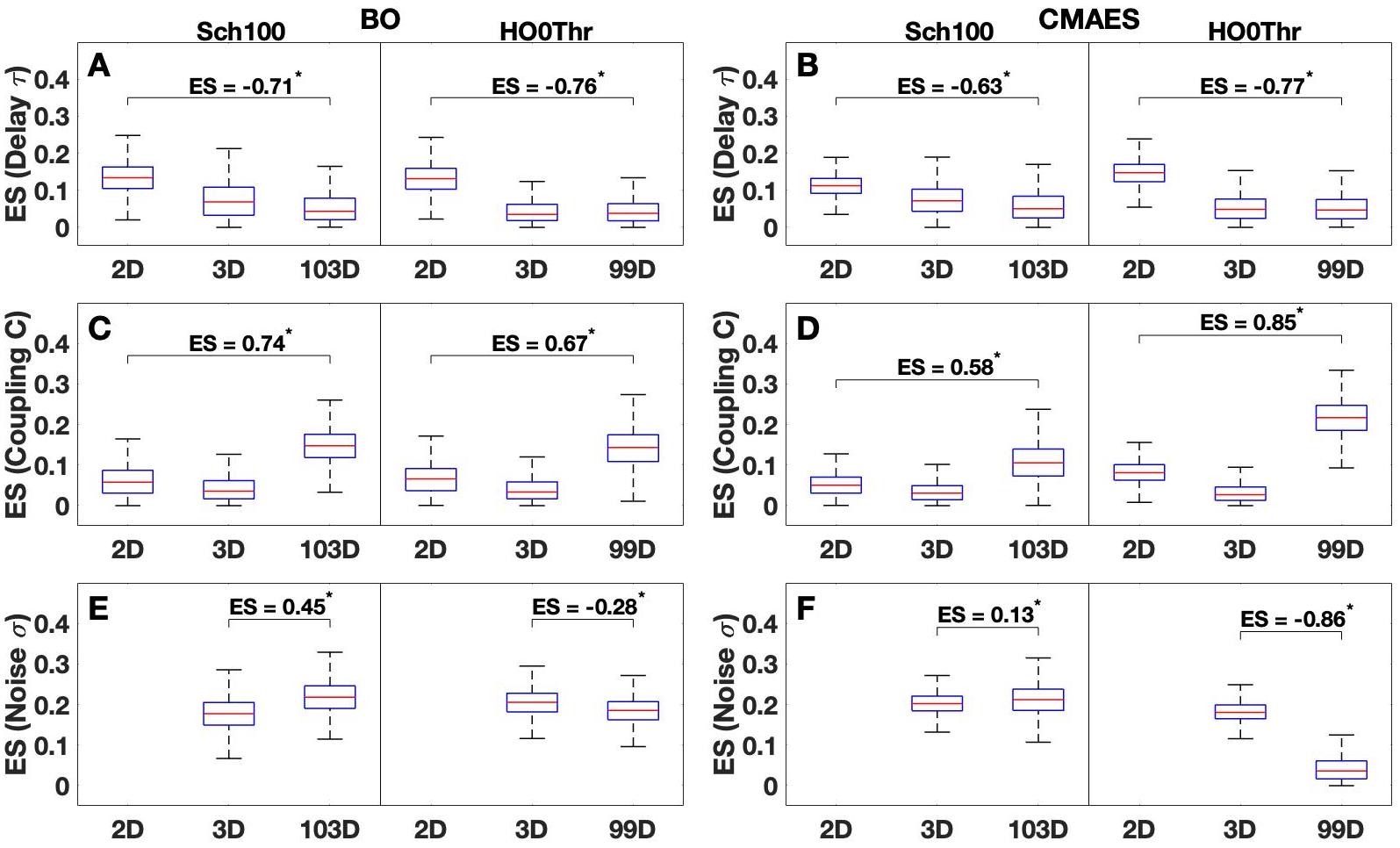 |
| --- |
| **Supplementary Fig 15. Sex differences in the optimized model parameters of delay** $\boldsymbol{\tau}$**, coupling** $\boldsymbol{C}$ **and noise intensity** $\boldsymbol{\sigma}$**.** Boxplots illustrate the distributions of the effect sizes (ES) for the sex differences in **(A-B)** delay $\tau$, **(C-D)** coupling $C$ and **(E-F)** noise intensity $\sigma$ observed during a random selection of one of the 30 available algorithm executions for every subject and a subsequent comparison of the respective parameter values across both groups (1000 repetitions). The utilized optimization methods and brain atlases are provided in the titles, while the considered parameter spaces are listed on the horizontal axes. On the vertical axes, the ES of the sex differences are depicted for the indicated model parameter. The changes (in ES) of the detected sex differences between the low- (2D, 3D) and the high-dimensional (99D/103D) cases are also indicated in the plots. Statistically significant changes (detected by the Wilcoxon rank-sum test) are marked with an asterisk. The significance level of 5%, $p$ < 0.05, has been Bonferroni-corrected for multiple comparisons. This figure was created with MATLAB R2021a ([www.mathworks.com](http://www.mathworks.com)). |

-----------------------------------------------------------------------------------------------------------------

| 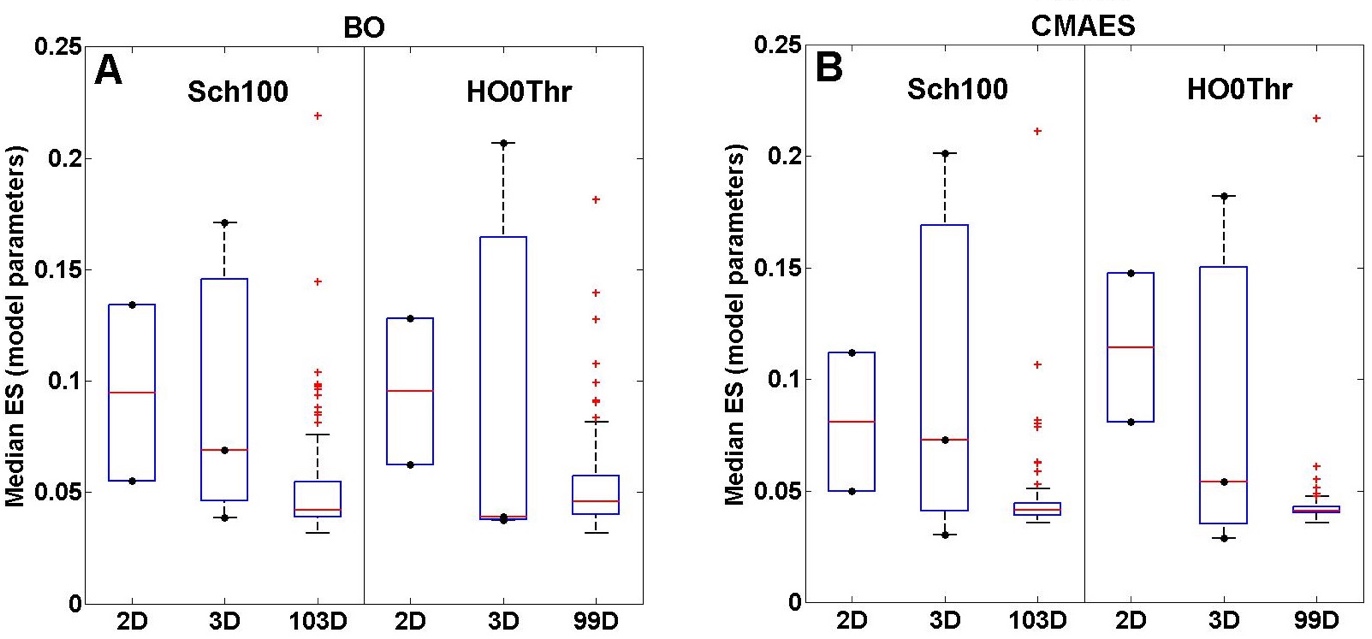 |
| --- |
| **Supplementary Fig 16.** **Median sex differences for all model parameters together.** For the optimization approaches based on **(A)** BO and **(B)** CMAES, boxplots visualize the distributions of the median effect sizes (ES) of the differences between males and females for all optimized model parameters. The medians were computed in analogy to the results presented in **Supplementary** **Fig 15**. In every considered parameter space, a random selection of one of the 30 available algorithm executions for every subject and a subsequent comparison of the respective parameter values across the groups of males and females were repeated 1000 times. This led to 1000 values of ES of sex differences for every model parameter in all considered parameter spaces. For the parameters of delay, coupling and noise intensity, these distributions are shown in **Supplementary Fig 15**. Here, only the medians of the individual distributions for every parameter were considered and assigned to the corresponding parameter space dimensions. In the low-dimensional cases (2D, 3D), the boxplots therefore depict only 2 or 3 values (for delay $\tau$, coupling $C$ and, additionally, the noise intensity $\sigma$). These are highlighted by black dots and correspond to the red lines presented in the boxplots in **Supplementary Fig 15**. The boxes corresponding to the high-dimensional cases (103D, 99D) illustrate the median extent of sex differences observable throughout all model parameters considered in this study, i.e., delay $\tau$, coupling $C$, noise intensity $\sigma$ and frequencies $\left( f_{i} \right)_{1\leq i\leq N}$. Solid vertical lines segregate the results for the Schaefer atlas (Sch100) and the Harvard-Oxford atlas (HO0Thr). This figure was created with MATLAB R2021a ([www.mathworks.com](http://www.mathworks.com)). |

-----------------------------------------------------------------------------------------------------------------

| **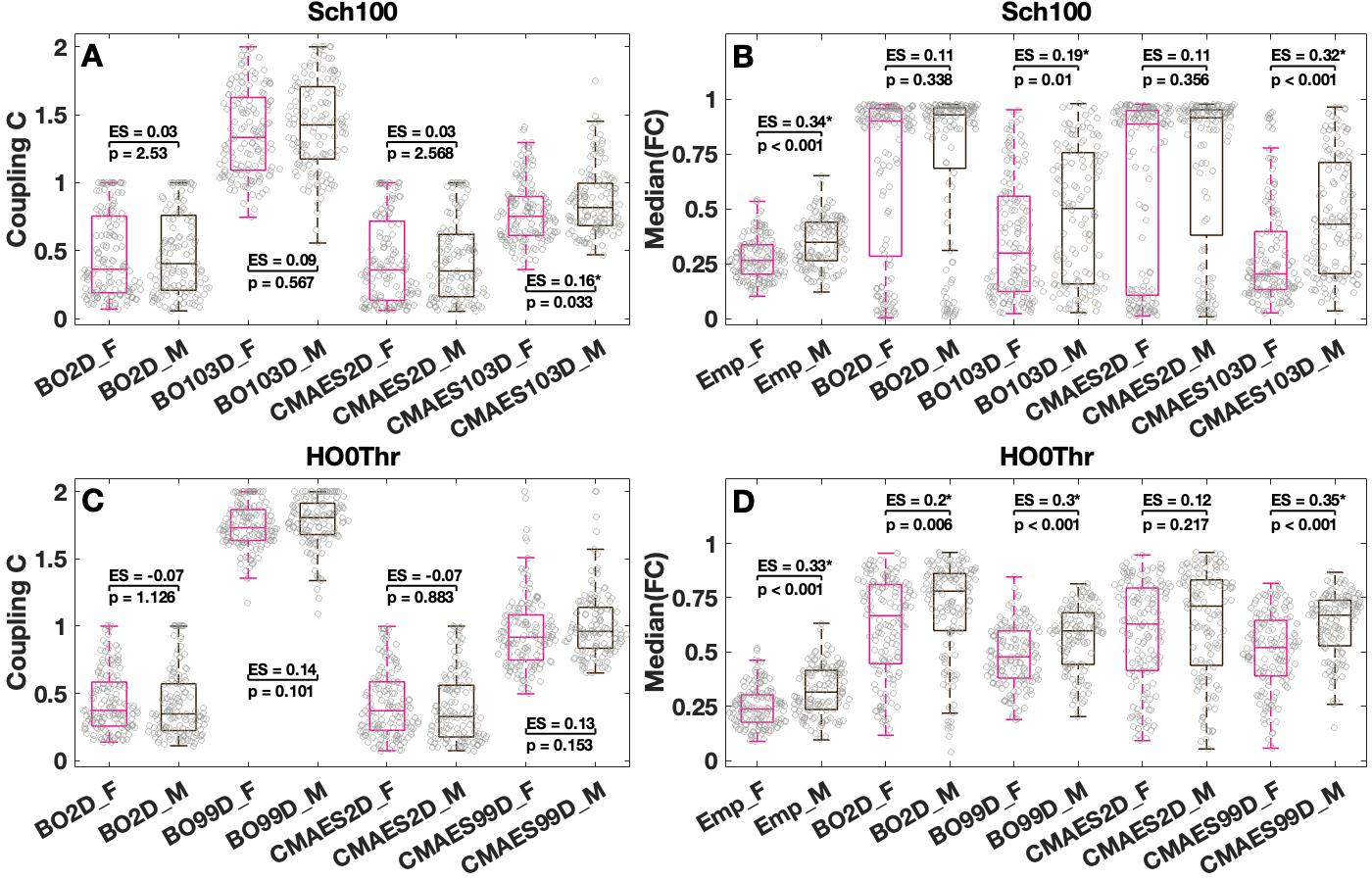** |
| --- |
| **Supplementary Fig 17. Distributions of median FC strengths and optimized coupling parameters for males and females. (A-D)** Similar to **Fig 7** of the main text. For every subject, the eFC from the concatenated sessions as well as the sFC matrix and coupling parameters yielding the median GoF across algorithm executions were selected (option 2, see Methods). This figure was created with MATLAB R2021a ([www.mathworks.com](http://www.mathworks.com)). |

-----------------------------------------------------------------------------------------------------------------

| **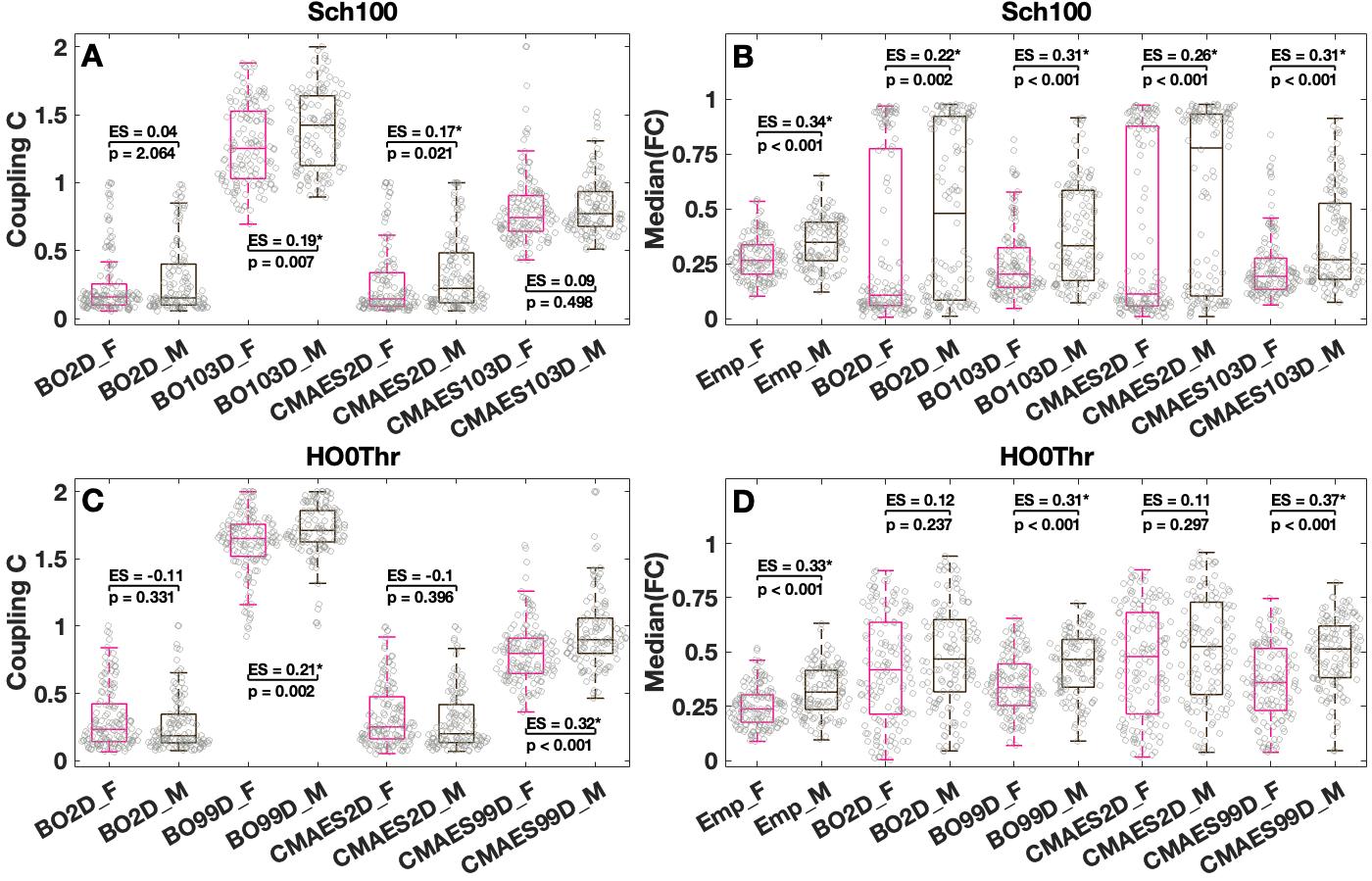** |
| --- |
| **Supplementary Fig 18. Distributions of median FC strengths and optimized coupling parameters for males and females. (A-D)** Similar to **Fig 7** of the main text. For every subject, the eFC from the concatenated sessions as well as the sFC matrix and coupling parameters yielding the minimal $\left\Vert\text{sFC}-\text{eFC} \right\Vert_{\text{F}}$ across algorithm executions were selected (option 3, see Methods). This figure was created with MATLAB R2021a ([www.mathworks.com](http://www.mathworks.com)). |

-----------------------------------------------------------------------------------------------------------------

| **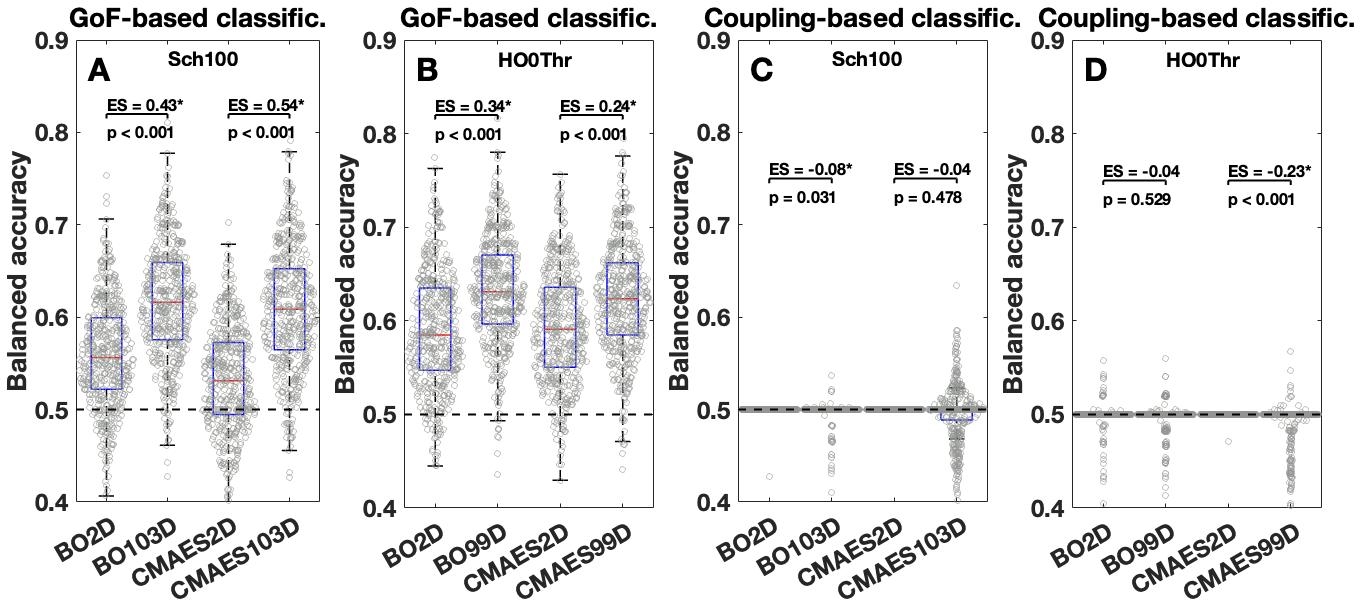** |
| --- |
| **Supplementary Fig 19. Model-based sex classification accuracies. (A-D)** Similar to **Fig 8** of the main text. For every subject, the GoF and parameter values derived from the algorithm execution yielding the median GoF were selected (option 2, see Methods). This figure was created with MATLAB R2021a ([www.mathworks.com](http://www.mathworks.com)). |

-----------------------------------------------------------------------------------------------------------------

| **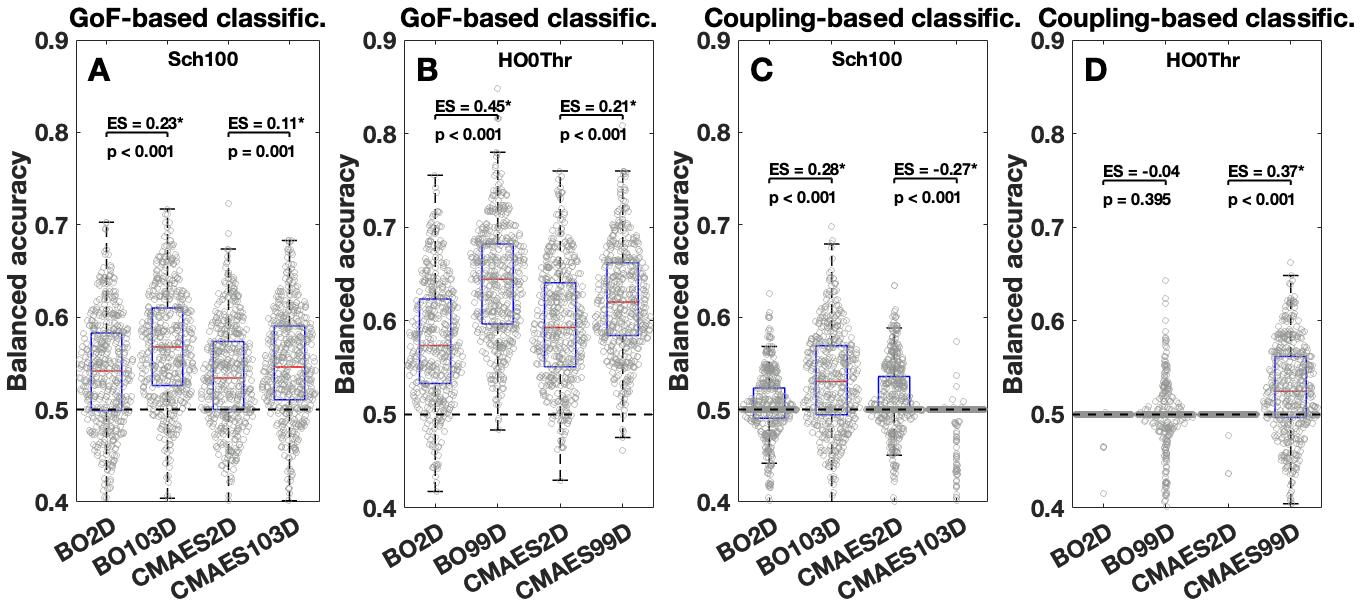** |
| --- |
| **Supplementary Fig 20. Model-based sex classification accuracies. (A-D)** Similar to **Fig 8** in the main text. For every subject, the GoF and parameter values derived from the algorithm execution yielding the minimal $\left\Vert\text{sFC}-\text{eFC} \right\Vert_{\text{F}}$ were selected (option 3, see Methods). This figure was created with MATLAB R2021a ([www.mathworks.com](http://www.mathworks.com)). |

**References**

1. Martinez-Cantin R. BayesOpt: A Bayesian Optimization Library for Nonlinear Optimization, Experimental Design and Bandits. Journal of Machine Learning Research. 2014;15:3735-9. doi: 10.48550/arXiv.1405.7430.

2. Wischnewski KJ, Eickhoff SB, Jirsa VK, Popovych OV. Towards an efficient validation of dynamical whole-brain models. Scientific Reports. 2022;12(1):4331. doi: 10.1038/s41598-022-07860-7.

3. Jülich-Supercomputing-Centre. JURECA: Data Centric and Booster Modules implementing the Modular Supercomputing Architecture at Jülich Supercomputing Centre. Journal of large-scale research facilities JLSRF. 2021;7. doi: 10.17815/jlsrf-7-182.

4. Hansen N. The CMA Evolution Strategy: A Tutorial. CoRR. 2016;abs/1604.00772. doi: 10.48550/arXiv.1604.00772.

5. Cicchetti DV, Sparrow SA. Developing criteria for establishing interrater reliability of specific items: applications to assessment of adaptive behavior. Am J Ment Defic. 1981;86(2):127-37. doi: PMID7315877.

6. Cicchetti DV. Guidelines, criteria, and rules of thumb for evaluating normed and standardized assessment instruments in psychology. Psychological Assessment. 1994;6(4):284-90. doi: 10.1037/1040-3590.6.4.284.

7. Amico E, Goni J. The quest for identifiability in human functional connectomes. Sci Rep. 2018;8(1):8254. doi: 10.1038/s41598-018-25089-1.

8. Zimmermann J, Griffiths J, Schirner M, Ritter P, McIntosh AR. Subject specificity of the correlation between large-scale structural and functional connectivity. Netw Neurosci. 2019;3(1):90-106. doi: 10.1162/netn_a_00055.

9. Domhof JWM, Eickhoff SB, Popovych OV. Reliability and subject specificity of personalized whole-brain dynamical models. Neuroimage. 2022;257:119321. doi: 10.1016/j.neuroimage.2022.119321.

10. Corey DM, Dunlap WP, Burke MJ. Averaging Correlations: Expected Values and Bias in Combined Pearsonrs and Fisher'szTransformations. The Journal of General Psychology. 1998;125(3):245-61. doi: 10.1080/00221309809595548.

11. Rosenthal R. Parametric Measures of Effect Size. In: Cooper H, Hedges LV, Valentine JC, editors. The Handbook of Research Synthesis. New York: Russel Sage Foundation; 1994. p. 231-44.

12. Devore JL, Peck R. Case Event Data and AE Using Descriptive Statistics. In: Bartz AE, editor. Basic Statistical Concepts. New York: Macmillan; 1988.
